# Rab7A Is Required for Efficient Production of Infectious HIV-1

**DOI:** 10.1371/journal.ppat.1002347

**Published:** 2011-11-03

**Authors:** Marina Caillet, Katy Janvier, Annegret Pelchen–Matthews, Delphine Delcroix-Genête, Grégory Camus, Mark Marsh, Clarisse Berlioz-Torrent

**Affiliations:** 1 INSERM, U1016, Institut Cochin, Paris, France; 2 CNRS, UMR8104, Paris, France; 3 Université Paris Descartes, Sorbonne Paris Cité, Paris, France; 4 Cell Biology Unit, MRC Laboratory for Molecular Cell Biology, University College London, London, United Kingdom; Universitätsklinikum Heidelberg, Germany

## Abstract

Retroviruses take advantage of cellular trafficking machineries to assemble and release new infectious particles. Rab proteins regulate specific steps in intracellular membrane trafficking by recruiting tethering, docking and fusion factors, as well as the actin- and microtubule-based motor proteins that facilitate vesicle traffic. Using virological tests and RNA interference targeting Rab proteins, we demonstrate that the late endosome-associated Rab7A is required for HIV-1 propagation. Analysis of the late steps of the HIV infection cycle shows that Rab7A regulates Env processing, the incorporation of mature Env glycoproteins into viral particles and HIV-1 infectivity. We also show that siRNA-mediated Rab7A depletion induces a BST2/Tetherin phenotype on HIV-1 release. BST2/Tetherin is a restriction factor that impedes HIV-1 release by tethering mature virus particles to the plasma membrane. Our results suggest that Rab7A contributes to the mechanism by which Vpu counteracts the restriction factor BST2/Tetherin and rescues HIV-1 release. Altogether, our results highlight new roles for a major regulator of the late endocytic pathway, Rab7A, in the late stages of the HIV-1 replication cycle.

## Author Summary

Human immunodeficiency virus (HIV) propagation requires the assistance of host cell factors at all stages of the infection cycle. HIV exploits components of the cellular membrane sorting machinery for its assembly, budding and release. Rab GTPases are key regulators of membrane-trafficking events, including exocytosis and endocytosis, in eukaryotic cells. Here we show that the late endosome associated Rab7A plays a major role in HIV-1 replication. We find that Rab7A regulates the production of infectious HIV-1 particles at two critical stages. First, Rab7A is required for efficient Env processing and, thus, for the incorporation of mature HIV-1 envelope glycoproteins into virions. Second, Rab7A contributes to the mechanism that counteracts the restriction imposed on HIV-1 release by the cellular restriction factor BST2/Tetherin that physically tethers viral particles to the plasma membrane of infected cells. Altogether these data highlight new roles for a major player of the late endocytic pathway, Rab7A, in the late stages of the HIV-1 replication cycle.

## Introduction

Human immunodeficiency virus type 1 (HIV-1) assembly, budding and release involves a highly orchestrated series of interactions between proteins encoded by the virus, viral genomic RNA and key cellular components of the cellular membrane sorting machineries [1]–[5]. These late steps of the viral replication cycle are coordinated by the viral Pr55 Gag precursor protein and are initiated by the binding of Gag complexes to the cytosolic face of the plasma membrane. This docking is regulated by the exposure of a myristoyl moiety that is co-translationally coupled to the Matrix (MA) domain of Gag, and by interaction of MA with phosphatidylinositol 4,5 bisphosphate [PI(4,5)P_2_] [6], [7]. Vesicular trafficking components, such as the clathrin adaptor protein (AP) complexes, the Golgi-localized γ-ear containing Arf-binding (GGA) and ADP ribosylation factor (ARF) proteins have also been implicated in Gag trafficking and virus release [8]. The AP-1 and AP-3 adaptor complexes, which normally select the cargoes carried by clathrin-coated vesicles, interact with Gag and appear to participate in its trafficking and in virus release [9]–[11]. Similarly, ARF proteins, key regulators of intracellular trafficking, support Gag trafficking to the plasma membrane whereas the GGA proteins, monomeric clathrin-binding factors regulating the sorting of mannose 6-phosphate receptor (MPR) from the TGN to endosomes, negatively regulate the production of virus particles [12]. In addition, transport machineries, including the AP-1 and AP-2 adaptor complexes [13]–[17] and TIP47 (tail-interacting protein of 47 kDa) [18]–[20] are involved in trafficking of the HIV-1 envelope glycoprotein (Env) and its incorporation into virions.

For scission, nascent viral particles hijack the ESCRT machinery (Endosomal Sorting Complexes Required for Transport) which normally functions in cytokinesis [21], [22], multi-vesicular body (MVB) formation and the targeting of ubiquitinated cargoes to the intralumenal vesicles of MVB [23]. Gag recruits TSG101, a component of ESCRT-I, or the ESCRT-associated protein AIP-1/ALIX through short peptide motifs in its C-terminal p6 domain, and this allows the recruitment of ESCRT-III complexes to promote the budding and scission of HIV-1 particles [24]–[27].

Following Gag-ESCRT-mediated viral particle scission, the accessory protein Vpu of HIV-1 promotes the release of mature viral particles by counteracting the action of the newly identified cellular restriction factor BST2/Tetherin (bone marrow stromal cell antigen 2, also called CD317/HM1.24) that impedes the release of fully assembled HIV-1 particles by physically tethering them to the cell surface. Vpu counteracts this restriction by downregulating BST2 [28], [29]. Interestingly, we recently showed that HRS (also called hepatocyte growth factor-regulated tyrosine kinase substrate [HGS]), a component of the ESCRT-0 complex, is required for Vpu to efficiently modulate BST2 expression and promote HIV-1 release, highlighting an additional role of the ESCRT machinery in virus production [30].

Rab GTPases are key regulators of membrane-trafficking events, including exocytosis and endocytosis, in eukaryotic cells. To identify additional cellular components required for HIV-1 formation, we explored the role of eight ubiquitously expressed Rab proteins (Rab1A, Rab4A, Rab5A, Rab6A, Rab7A, Rab8A, Rab9A, Rab11A) involved in the endocytic and exocytic pathways. Each of these proteins localizes to distinct intracellular compartments and regulates specific steps of vesicle trafficking by recruiting tethering, docking and fusion factors as well as actin- or microtubule-based motor proteins [31]–[34]. Using specific RNA interference targeting Rab proteins, and virological assays, we demonstrate that Rab7A is required for efficient HIV-1 propagation. Rab7A plays an important role in the organization of late endocytic compartments [35]–[42], and in the maturation of late endosomes and phagosomes and their fusion with lysosomes [43]–[46]. Recent studies have shown that Rab7A induces the recruitment of dynein and dynactin motors and regulates transport toward the minus-end of microtubules [47]. Analysis of the later stages of HIV-1 replication shows that Rab7A is required for efficient HIV-1 release and infectivity. We show that Rab7A depletion reduces Env processing, increasing the levels of the uncleaved Env precursor gp160 in virus-producing cells and in virus particles, thereby impairing viral infectivity. Moreover, Rab7A knockdown causes an accumulation of virus particles at the surface of infected cells, an effect related to the expression of the restriction factor BST2/Tetherin. We find that Rab7A participates in the mechanism by which Vpu counteracts BST2/Tetherin, thereby promoting HIV-1 release. Altogether, our results show that Rab7A is a key regulator in the late stages of the HIV-1 infection cycle, influencing several steps in the production and release of infectious viral particles.

## Results

### Rab7A is essential for HIV-1 propagation

**Figure 1:**
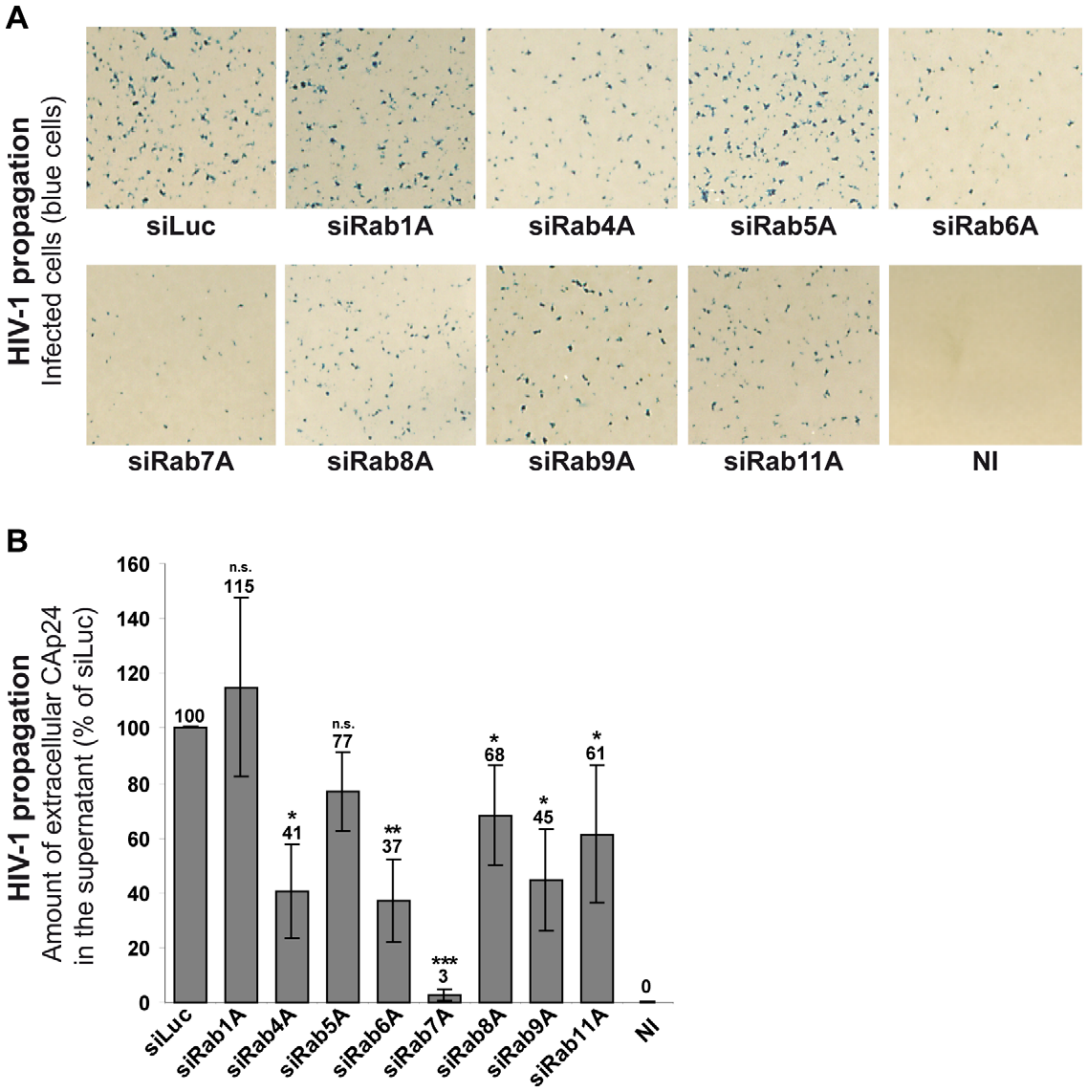
Rab7A depletion reduces HIV-1 propagation. HeLa P4-R5 MAGI cells transfected with either siRNA control Luciferase (siLuc) or siRNAs targeting Rab proteins (siRab) were not infected (NI) or infected with wild type HIV-1 NL4-3 (HIV-1) at a low MOI (0.005), and left for 4 days to allow viral propagation. (**A**) X-Gal staining of infected cell cultures. The blue staining corresponds to infected cells after multiple rounds of HIV-1 infection. This experiment is representative of 3 independent experiments performed in duplicate. (**B**) HIV-1 propagation was scored by measuring the amount of HIV-1 CAp24 released into the culture supernatants by ELISA quantification. The amounts of released CAp24 were normalized to those obtained for the control cells, set as 100%. Bars represent the means of percentage values ± SD for 3 independent experiments. *P* values were calculated based on *t-*test. Significant results (***, p<0.001, **, p<0.01 and *, p<0.05) are indicated (n.s.  =  not significant).

To explore the role of Rab proteins in the HIV-1 replication cycle, we analyzed the effect of Rab1A, Rab4A, Rab5A, Rab6A, Rab7A, Rab8A, Rab9A and Rab11A depletion on HIV-1 propagation in HeLa MAGI P4-R5 cells, which stably express the HIV-1 receptor/coreceptors CD4, CCR5 and CXCR4. These cells have the β-galactosidase reporter gene under the control of the HIV-1 LTR, allowing the detection of infected cells by X-gal staining (blue cells). HeLa P4-R5 cells were treated with siRNAs targeting the Rab proteins or control siRNA targeting the luciferase sequence (siLuc) (Figure S1A). Treated cells were then infected with HIV-1 NL4-3 strain (referred herein as HIV-1) at a multiplicity of infection (MOI) of 0.005 and left for 4 days to enable the virus to spread in the cultures. HIV-1 propagation was monitored by X-gal staining of infected cells (Figure 1A) and by quantification of the HIV capsid protein CAp24 released into the medium (Figure 1B). Depletion of Rab proteins was confirmed by western blot analysis of the cells. Silencing was efficient and specific as extinction of each Rab tested did not affect the expression of the others (Figure S1B). No significant alteration of HIV-1 replication was observed following depletion of Rab1A and Rab5A compared to the control cells treated with siRNA against luciferase (Figures 1A and 1B). A moderate decrease in HIV-1 replication was observed upon knockdown of Rab4A, Rab8A, Rab9A and Rab11A, with ∼30 to 60% less CAp24 detected in the supernatants of these cells compared to the control. The effects of Rab9A and Rab11A depletion were similar to those described by Murray and collaborators [48]. Moreover, consistent with a previous study [49], extinction of Rab6A expression reduced (>60% decrease) HIV-1 dissemination (Figures 1A and B). Interestingly, the most potent effect on HIV-1 propagation was observed in cells depleted of Rab7A. Indeed, four days post-infection only a few infected cells were observed in Rab7A-depleted cultures (Figure 1A) and the level of CAp24 in the cell supernatants was 30-fold lower than the control (Figure 1B). These results suggest a major role for Rab7A in HIV-1 propagation in HeLa P4-R5 cells.

### Rab7A is required for the release of infectious HIV-1 particles

To decipher the role of Rab7A in HIV-1 replication, we tested whether the reduction of HIV-1 propagation observed in Rab7A-depleted HeLa P4-R5 was a consequence of an alteration of HIV-1 entry into cells. HeLa P4-R5 cells were treated with siRNAs targeting Rab7A or luciferase (siLuc; negative control) and infected for 2 hours with HIV-1 at MOI = 0.005. The CXCR4 antagonist AMD3100 was added to the culture medium 2 hours post-infection to block re-infection of the cells. Entry of HIV-1 was quantified by counting the number of infected cells. A modest decrease of HIV-1 entry (∼35%) was observed in Rab7A-depleted cells compared to the control cells (Figure S2A). Similar results were obtained for HIV pseudotyped with VSV-G envelope (the virus used in the subsequent experiments; ∼30% decrease; Figure S2B), indicating that HIV-1 entry was not dramatically affected by Rab7A depletion. These data therefore cannot fully explain the dramatic reduction of HIV propagation observed in Rab7A-depleted cells (Figure 1).

**Figure 2:**
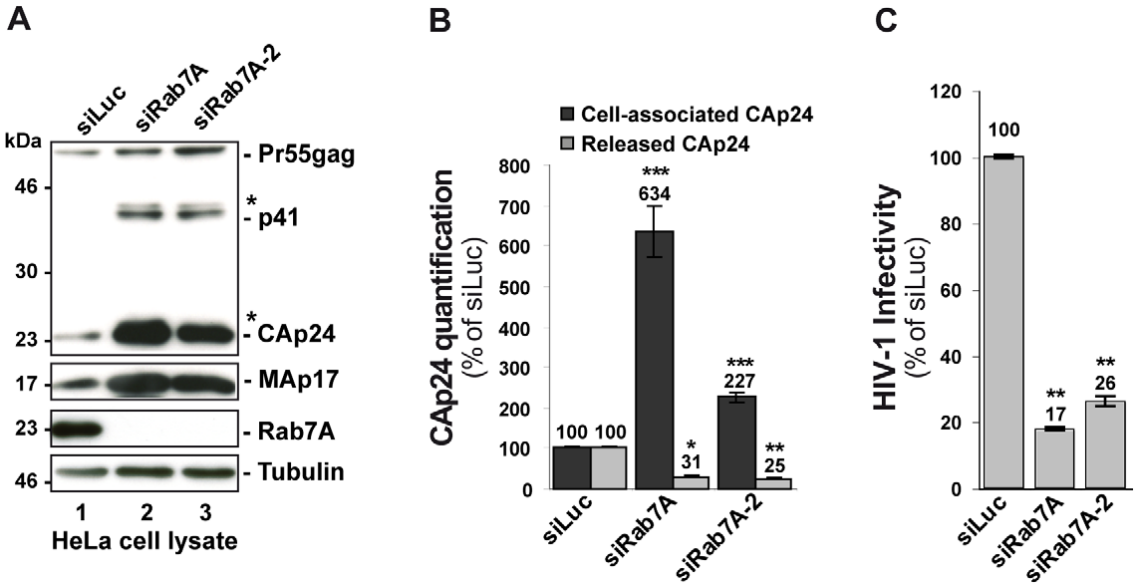
Rab7A depletion reduces the release of HIV-1 particles. HeLa cells transfected with either siRNA control Luciferase (siLuc) or siRNA targeting Rab7A proteins (siRab7A and siRab7A-2) were infected with the VSV-G-pseudotyped HIV-1. (**A**) Western blot analysis of infected siRNA-treated cells. Tubulin is the loading control. This western blot is representative of 3 independent experiments. (**B**) The amounts of cell associated (Cell-associated CAp24, black graph bars) and released HIV-1 CAp24 (Released CAp24, grey graph bars), measured by ELISA for cell cultures depleted for Rab7A or Rab7A-2 were normalized to those obtained for the control cells, set as 100%. Bars represent the mean of percentage values ± SD for 3 independent experiments. (**C**) Infectivity of HIV-1 released from the cells. Virus titres were normalized for the quantity of released CAp24 (Infectious units/µg CAp24) and compared to the infectivity of virus from control cells, set as 100%. Bars represent the means of percentage values ± SD for 3 independent experiments. (**B-C**) *P* values were calculated based on *t-*test. Significant results (***, p<0.001, **, p<0.01 and *, p<0.05) are indicated.

We next investigated whether Rab7A is involved in the late stages of HIV-1 replication, corresponding to the budding and release of infectious particles. HeLa cells were transfected with siRNA against Rab7A or control siRNA, and infected with HIV-1 NL4-3 pseudotyped with VSV-G envelope at a MOI of 0.5. VSV-G pseudotyping enabled us to monitor HIV-1 production after a single round of infection in HeLa cells. Twenty-four hours after infection, the impact of Rab7A depletion on the production of infectious HIV-1 was assessed. To better validate our siRNA experiments, two distinct siRNAs against Rab7A (referred to as siRab7A and siRab7A-2) were used. Rab7A expression was checked by western blotting (Figure 2A). The expression level and processing of Gag in the cells was also analyzed by western blotting, and virus release was monitored by ELISA quantification of the processed Gag product CAp24. Western blot analysis revealed no major alteration of Gag processing upon Rab7A depletion but showed a strong accumulation of Gag precursor and processed products (Gag p41, CAp24, MAp17) in Rab7A knockdown cells compared to the control (Figure 2A, compare lanes 2–3 to lane 1). Consistent with this observation, ELISA quantification of CAp24 showed a 2 to 6-fold increase in cell-associated CAp24 depending on the siRNA used (Figure 2B). The accumulation of cell-associated Gag, together with a ∼70% decrease of CAp24 released into the supernatant of Rab7A-depleted cells (Figure 2B), suggested that Rab7A is essential for efficient release of HIV-1 from HeLa cells.

We also scored the infectious titres of the particles by infection of HeLa TZM-bl indicator cells. The results were normalized to the amount of CAp24 present in the cell supernatants (referred herein as HIV-1 infectivity). Interestingly, viral particles produced from Rab7A knockdown cells were poorly infectious (70 to 80% less infectious compared to the control), showing that silencing of Rab7A not only reduces the release of viral particles, but also diminishes the infectivity of the HIV-1 particles that are released (Figure 2C).

### Rab7A is essential for HIV-1 infectivity

We investigated whether the reduced infectivity of virus particles produced from Rab7A-depleted cells might be due to an alteration of viral genomic RNA packaging into the budding virions, as it has been shown that HIV RNAs are transported on endosomal vesicles [50], [51]. The amount of viral genomic RNA (gRNA) present in the viral preparations from siRNA treated HeLa cells was measured by real time PCR (Figure S3D) and normalized to the amount of CAp24 released (Figure S3A). No variation of this ratio was observed (Figure S3F), indicating that Rab7A depletion does not perturb gRNA packaging into viral particles. Interestingly, higher levels of viral genomic RNA were detected in Rab7A-depleted producer cells compared to controls (Figure S3E). Along with the increase of cell-associated CAp24 (Figure S3B), these data suggest that Rab7A silencing induces the accumulation of assembled virus in the cells.

**Figure 3:**
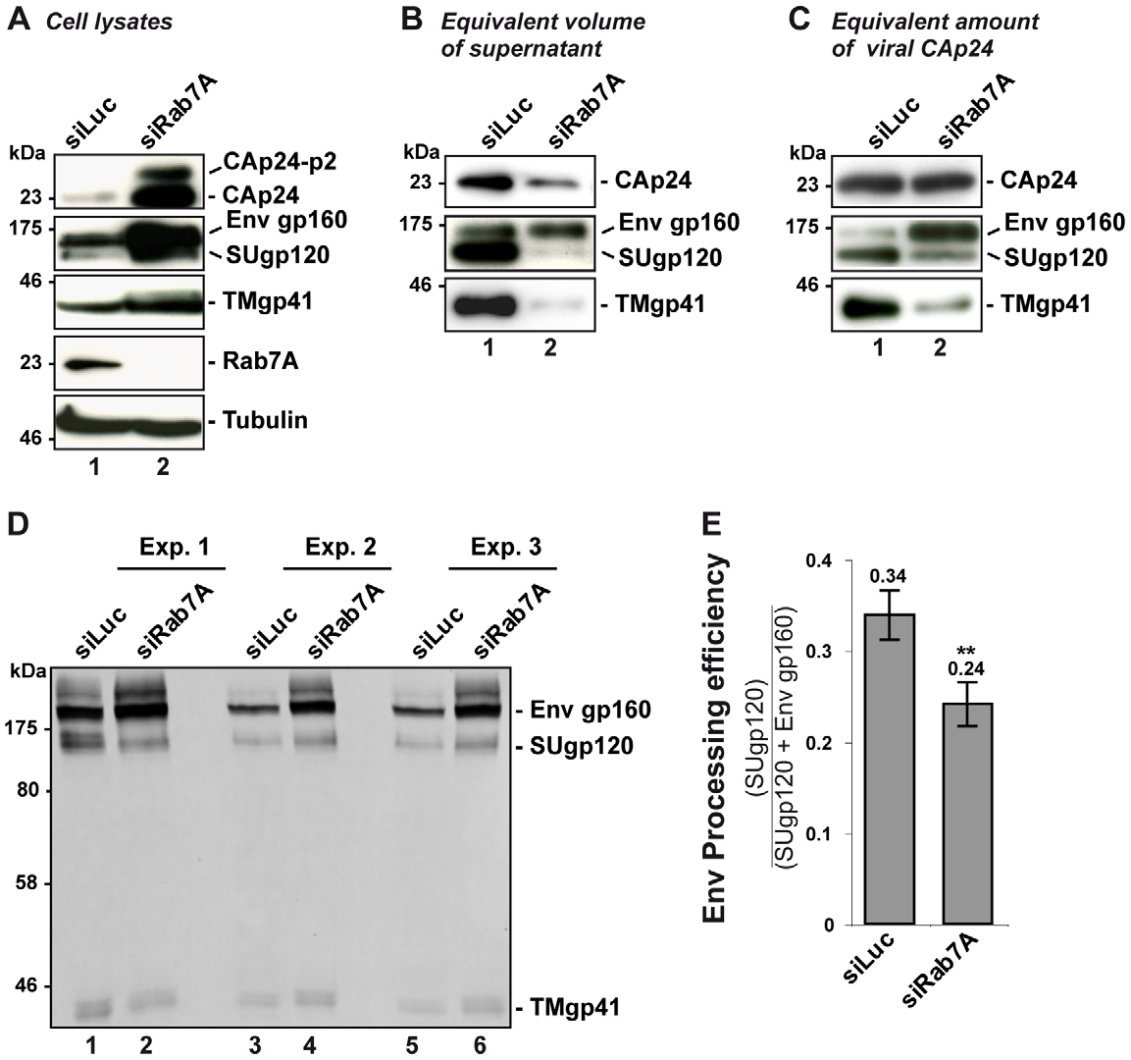
Rab7A depletion affects Env processing and the incorporation of mature Env into viral particles. HeLa cells transfected with either siRNA control Luciferase (siLuc) or siRNA targeting Rab7A (siRab7A) were infected with VSV-G pseudotyped wt NL4-3 HIV-1 (HIV-1 WT). This experiment is representative of 3 independent experiments done in duplicate. (**A**) Equal amounts of cell lysate (50 µg protein) were loaded to visualize the intracellular accumulation of viral proteins in Rab7A-depleted cells. (**B**) Equal volumes of the virus samples were loaded to visualize the decrease of virus released from Rab7A-depleted cells. (**C)** Equal amounts of CAp24 (30 µg) per lane were loaded to visualize Env content for a fixed amount of viral particles. (**D**) Further analysis of cell lysates. For three independent experiments (Exp. 1, 2, and 3)**,** 160 µg protein of control cell lysates or 80 µg protein of Rab7A-depleted cell lysates were loaded and Env products (Env gp160 precursor, SUgp120 and TMgp41) were detected using anti-SUgp120 (110H) and anti-TMgp41 (41A) antibodies. (**E**) Env signals were quantified for these 3 independent experiments and values obtained were used to calculate the Env processing efficiency (the ratio between the signals detected for the processed SUgp120 subunits relative to the total Env detected, gp160 + SUgp120. Bars represent the mean of the ratios ± SD for these 3 experiments. *P* values were calculated based on *t-*test. Significant results (**, p<0.01) are indicated.

To further characterize the impact of Rab7A depletion on HIV-1 infectivity, we analyzed the composition of viral particles produced by Rab7A-depleted HeLa cells by western blotting for Gag and Env. Western blot analysis of equal volumes of the viral preparation produced from siRNA-treated cells showed a decrease in the amount of Gag CAp24 and the Env proteins, TMgp41 and SUgp120, upon Rab7A depletion compared to the control (Figure 3B), consistent with the decrease in viral particle release shown in Figure 2.

Surprisingly, similar levels of immature Env precursor (gp160) were detected in both viral samples, suggesting that Rab7A knockdown may affect Env processing in the infected cells (Figure 3B). To investigate this, equivalent amounts of virus particles were loaded (30 µg) and the Env content was analyzed by western blotting (Figure 3C). Here, compared to the control, little processed SUgp120 and TMgp41 was detected in the virus particles produced by the Rab7A-depleted cells. By contrast, higher levels of gp160 were seen in these viruses. Increased amounts of gp160 and the mature products, SUgp120 and TMgp41, were also detected in the corresponding cell lysates, suggesting that the reduced levels of mature Env observed in the virions were not due to the absence of these Env subunits in the producer cells (Figure 3A). We further investigated Env processing in infected cell lysates. Control cell lysates (160 µg) or lysates from Rab7A-depleted cells (80 µg was loaded to avoid saturating the Env signal in Rab7A knockdown samples) were analyzed and the intensities of the gp160 and SUgp120 bands were quantified. Env processing efficiency was determined by calculating the ratio between the signal of SUgp120 to those of SUgp120 + Env gp160 (Figures 3D and 3E). This revealed a lower efficiency of Env processing in Rab7A knockdown cells (Figure 3E), which explains the presence of mainly immature Env gp160 in the virus particles and thus the decreased infectivity of the virus released from Rab7A knockdown cells (Figure 2C and S3C).

Together, these data show that Rab7A silencing leads to a reduction in production of HIV-1 particles. As these particles contain the Env precursor gp160 and reduced levels of mature Env, their infectivity is reduced. Thus, Rab7A is required for the efficient production of infectious HIV-1.

### Rab7A depletion induces the accumulation of mature viruses at the cell surface

**Figure 4:**
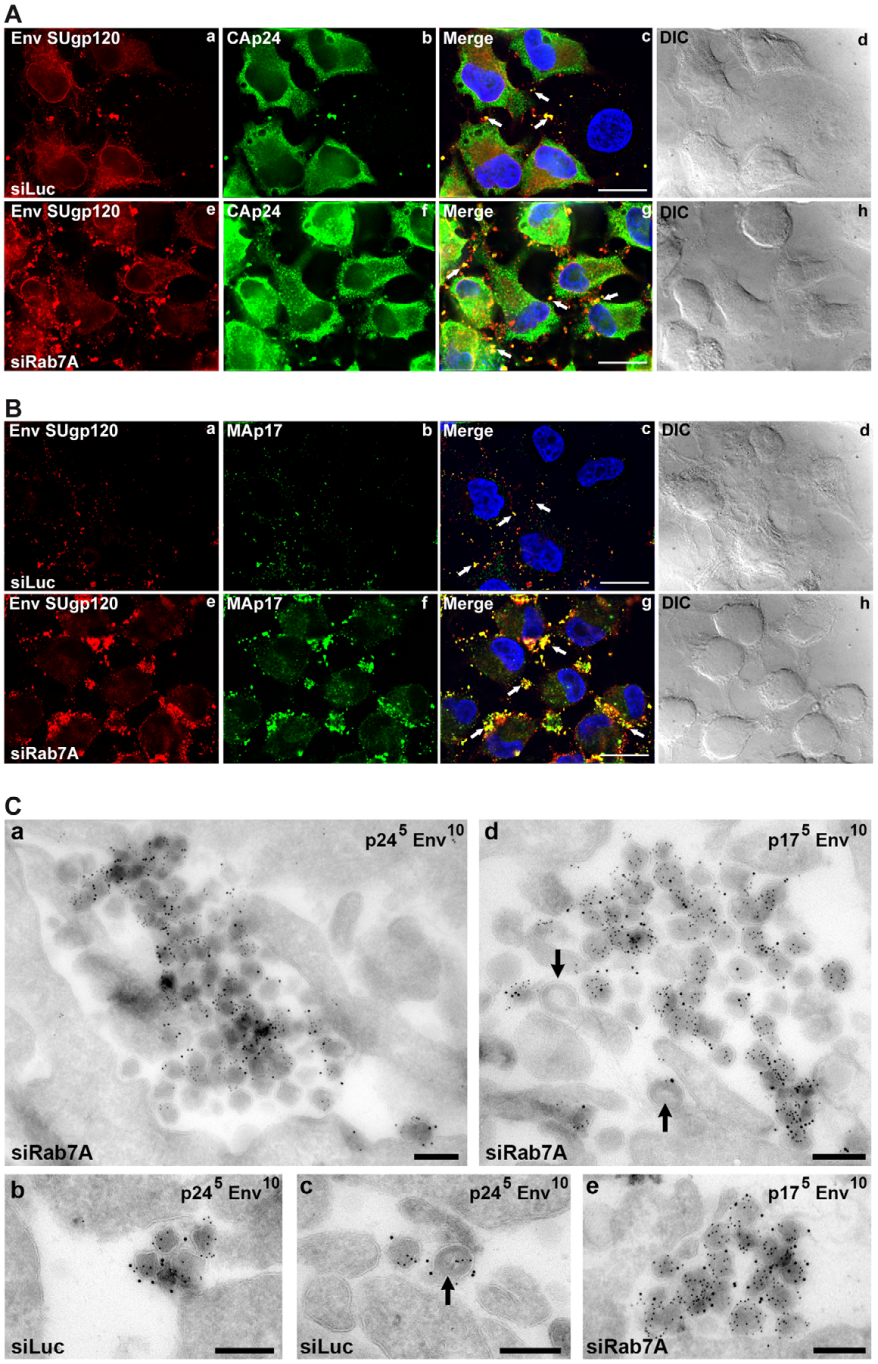
Silencing of Rab7A induces accumulation of virus clusters at the cell surface. HeLa cells transfected with either siRNA control Luciferase (siLuc) or siRNA targeting Rab7A (siRab7A) were infected with VSV-G-pseudotyped HIV-1 and analyzed 24 h after infection. (**A**) Cells were fixed with PFA and immunolabelled with anti-CAp24 (CAp24) and anti-Env (Env SUgp120) antibodies. (**B**) Living cells were stained at 4°C with anti-Env antibody (Env SUgp120) and appropriate fluorophore-conjugated secondary antibody. Cells were then fixed with PFA, permeabilized and labelled with anti-MAp17 (MAp17) antibody. White arrows show spots co-labelling for Env and CAp24 or MAp17 at the cell surface. Scale bars = 20 µm. (**C**) Cells were fixed and processed for EM immunolabelling. Ultrathin cryosections were double labelled for Env with 10 nm protein A-gold particles and for either CAp24 (panels a, b, c) or MAp17 (panels d and e) and 5 nm protein A-gold. Virus particles were detected at the cell surface. Black arrows mark immature viruses and buds. Scale bars = 200 nm.

Since the previous experiments indicated that Rab7A depletion may inhibit HIV-1 release from infected cells, siRNA-treated HeLa cells infected with VSV-G pseudotyped NL4-3 HIV-1 were analyzed by immunofluorescence (Figure 4A and 4B). Labelling with antibodies directed against the Env SUgp120 subunit and CAp24 (detecting the CA sub-product and the Gag precursor) showed numerous infected cells with small spots that co-stained for Gag and Env. Staining of Rab7A knockdown cells showed an increased number of larger, more prominent spots co-labelled with anti-Env and anti-CA antibodies at the periphery of the cells compared to the control (see white arrows on Figure 4A, compare panels c and g). Moreover, the intracellular staining of Env and Gag also appeared more intense in Rab7A-depleted cells (Figure 4A, panels e-h), which correlates with the increased amount of cell-associated Gag and Env detected by western blotting (see Figures 2A and 3A).

In order to determine whether the viral products accumulated at the periphery of the cells were present at the cell surface and corresponded to mature viruses, non-permeabilized cells were stained for cell surface Env, then fixed, permeabilized and stained with anti-MAp17, that detects only the cleaved Gag product MAp17 and thus mature viruses. As shown on Figure 4B, Env labelling present at the cell surface was increased upon Rab7A knockdown (compare panels a and e) and mostly co-localized with MAp17 labelling present in large spots located at cell peripheries (panel f). The increased number of spots co-labelled with MA and Env at the cell surface of Rab7A-depleted cells (see white arrows) suggests that Rab7A silencing may induce the accumulation of mature viruses at the cell surface.

To investigate directly whether depletion of Rab7A leads to the accumulation of virus particles at the cell surface, ultrathin cryosections from infected cells were immunolabelled for CAp24 or MAp17 and Env and examined by electron microscopy (Figure 4C). Large clusters of Env-containing virus particles were observed at the surface of Rab7A-depleted cells (Figure 4C, panels a, d and e), while on control cells we usually only saw single virus particles or small groups of labelled virions (Figure 4C, panels b and c). Most virus particles had a mature morphology and stained strongly with the antibody 4C9, which detects only the cleaved mature MAp17 and therefore labels primarily mature virus particles, though some immature virus particles and budding figures were also observed (black arrows in Figure 4C, panels c and d, see also Figure S4). Together, these data show that Rab7A silencing induces the accumulation of virus clusters at the surface of HIV-1 infected cells.

### Reduced HIV-1 release in Rab7A-depleted cells is related to expression of the restriction factor BST2/Tetherin

The accumulation of mature viral particles at the surface of infected cells, and decreased release of viral particles, are reminiscent of the phenotype described in BST2-expressing cells infected with Vpu-deficient HIV-1 strains. BST2 has been shown to physically tether newly formed virus particles at the surface of infected cells, preventing their release. Vpu relieves this restriction by promoting the degradation of BST2 and downregulating its cell surface expression [28]–[30], [52]–[56].

**Figure 5:**
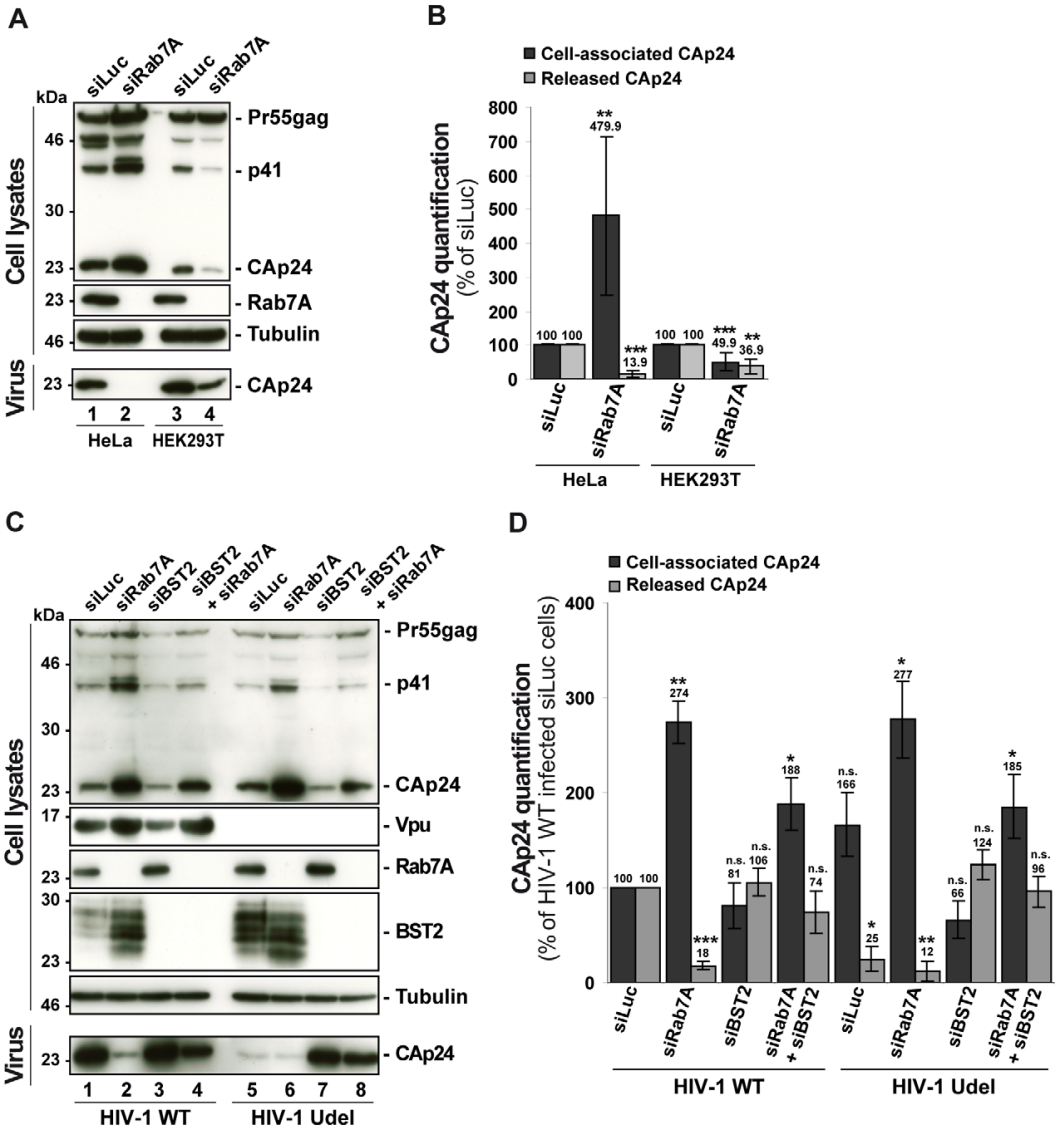
Reduced HIV-1 release after depletion of Rab7A is related to expression of the restriction factor BST2. (**A**–**B**) Effect of Rab7A depletion on the release of HIV-1 particles from non-restrictive HEK 293T cells. HeLa cells and HEK 293T cells transfected with either control siRNA (siLuc) or siRNA targeting Rab7A were infected with VSV-G-pseudotyped HIV-1. (**A**) Western blot analysis of infected siRNA-treated cells (Upper panels) and viral particles released into the supernatant (Lower panel)**.** Tubulin is the loading control. This western blot is representative of 3 independent experiments done in duplicate. (**B**) CAp24 levels present within the cells (Cell-associated CAp24, dark grey graph bars) and released from the infected cells (Released CAp24, light-grey graph bars), measured by ELISA quantification for Rab7A-depleted cells, were normalized to those obtained for the control cells, set as 100%. Bars represent the mean of percentage values ± SD from 3 independent experiments. *P* values were calculated based on *t-*test. Significant results (***, p<0.001 and **, p<0.01) are indicated. (**C-D**) Effect of Rab7A and BST2 depletion on the release of HIV-1 particles from restrictive HeLa cells. HeLa cells were transfected with either control siRNA Luciferase (siLuc) (lanes 1 and 5) or siRNA targeting Rab7A protein (siRab7A) (lanes 2 and 6), BST2 (siBST2) (lanes 3 and 7) or both Rab7A and BST2 (lanes 4 and 8). Cells were then infected with either VSV-G pseudotyped wt NL4-3 HIV-1 (HIV-1 WT) viruses or VSV-G pseudotyped Vpu-defective NL4-3 (HIV-1 Udel) viruses. (**C**) Western blot analysis of infected siRNA-treated cells. Tubulin is the control loading. Equal volumes of the viral samples were loaded to visualize the partial rescue of virus release in Rab7A/BST2-depleted cells. The western blot is representative of 3 independent experiments done in duplicate. (**D**) HIV-1 CAp24 present within the cells (Cell-associated CAp24, black graph bars) and CAp24 released into the supernatant of the infected cells (Released CAp24, grey graphs bars), measured by ELISA quantification for Rab7A and/or BST2-depleted cells infected with wt or Udel HIV-1, were normalized to those of the control cells infected with wt HIV-1, set to 100%. Bars represent the means of percentage values ± SD for 3 independent experiments. *P* values were calculated based on *t-*test. Significant results (***, p<0.001, **, p<0.01 and *, p<0.05) are indicated (n.s.  =  not significant).

To investigate whether the effect of Rab7A depletion on HIV-1 release is related to Vpu-induced BST2 downregulation, we compared the effect of Rab7A depletion on HIV-1 production in restrictive HeLa cells and in HEK 293T, which do not normally express BST2 and are referred to as “non-restrictive” cells [28], [29]. RNAi-mediated Rab7A knockdown was similar in HEK 293T and HeLa cells (> 95%; Figure 5A). In HeLa cells (Figures 5A and 5B), Rab7A depletion inhibited the release of HIV-1 and induced an accumulation of cell-associated CAp24 as shown before. In HEK 293T cells, a reduced amount of CAp24 was detected in the supernatant. However, this was paralleled by a decrease in the amount of cell-associated CAp24 in Rab7A knockdown HEK 293T cells (Figures 5A and 5B), suggesting that the decreased CAp24 release observed in HEK 293T cultures most likely reflects lower levels of cell-associated CAp24 rather than a defect in virus release (Figure 5B).

We also analyzed the outcome of Rab7A silencing on the production of wt and Vpu-defective HIV-1 from HeLa cells with or without BST2. HeLa cells were transfected with control siRNA or siRNAs targeting Rab7A and BST2 alone or together and subsequently infected with either VSV-G pseudotyped wt NL4-3 HIV-1 (HIV-1 WT) or VSV-G pseudotyped Vpu-defective NL4-3 HIV-1 (HIV Udel) at an equivalent MOI (MOI = 0.5). As previously reported [28], [29], [57] in HeLa cells, HIV-1 release is less efficient in the absence of Vpu; less CAp24 was released into the supernatant of HIV-1 Udel-infected cells (Figures 5C and 5D, compare lanes 1 and 5). Interestingly, Rab7A depletion reduced release of both WT and Udel viruses and increased the level of cell-associated Gag products (Figures 5C and 5D, compare lanes 1 and 2 with lanes 5 and 6). As previously described [28], [29], the release of Vpu-defective HIV-1 was restored in cells depleted for BST2, as higher amounts of Udel virus were produced from these cells compared to control cells (Figures 5C and 5D, compare lanes 5 and 7). Interestingly, wt and Udel HIV-1 release was less affected by Rab7A depletion in HeLa cells devoid of BST2 (Figure 5C, compare CAp24 released, lanes 2 and 4, and lanes 6 and 8), indicating that the impact of Rab7A on HIV-1 release may be related to BST2. However, we noted that the level of cell-associated Gag in Rab7A and BST2-depleted cells, although lower than in Rab7A knockdown cells, remains higher than the level detected in cells depleted for BST2 alone. Thus, the striking increase in cell-associated Gag observed in Rab7A-depleted cells may result not only from BST2-induced retention of viral particles, but also from additional functions of Rab7A on virus release. Nevertheless, our results indicate that, at least in part, the effect of Rab7A knockdown on HIV-1 release is related to BST2 expression in the producer cells.

To further characterize the impact of Rab7A depletion on Vpu-induced downregulation of BST2, we analyzed the steady state level of BST2 (Figure 5C) in siRNA treated-infected cells by western blotting. As previously described [30], [52], [53], [56], HIV-1 Vpu reduced BST2 levels in infected cells (Figure 5C, compare lanes 1 and 5). Interestingly, downregulation of BST2 expression by Vpu was abrogated by Rab7A depletion.

### Rab7A regulates the constitutive turnover of BST2

**Figure 6:**
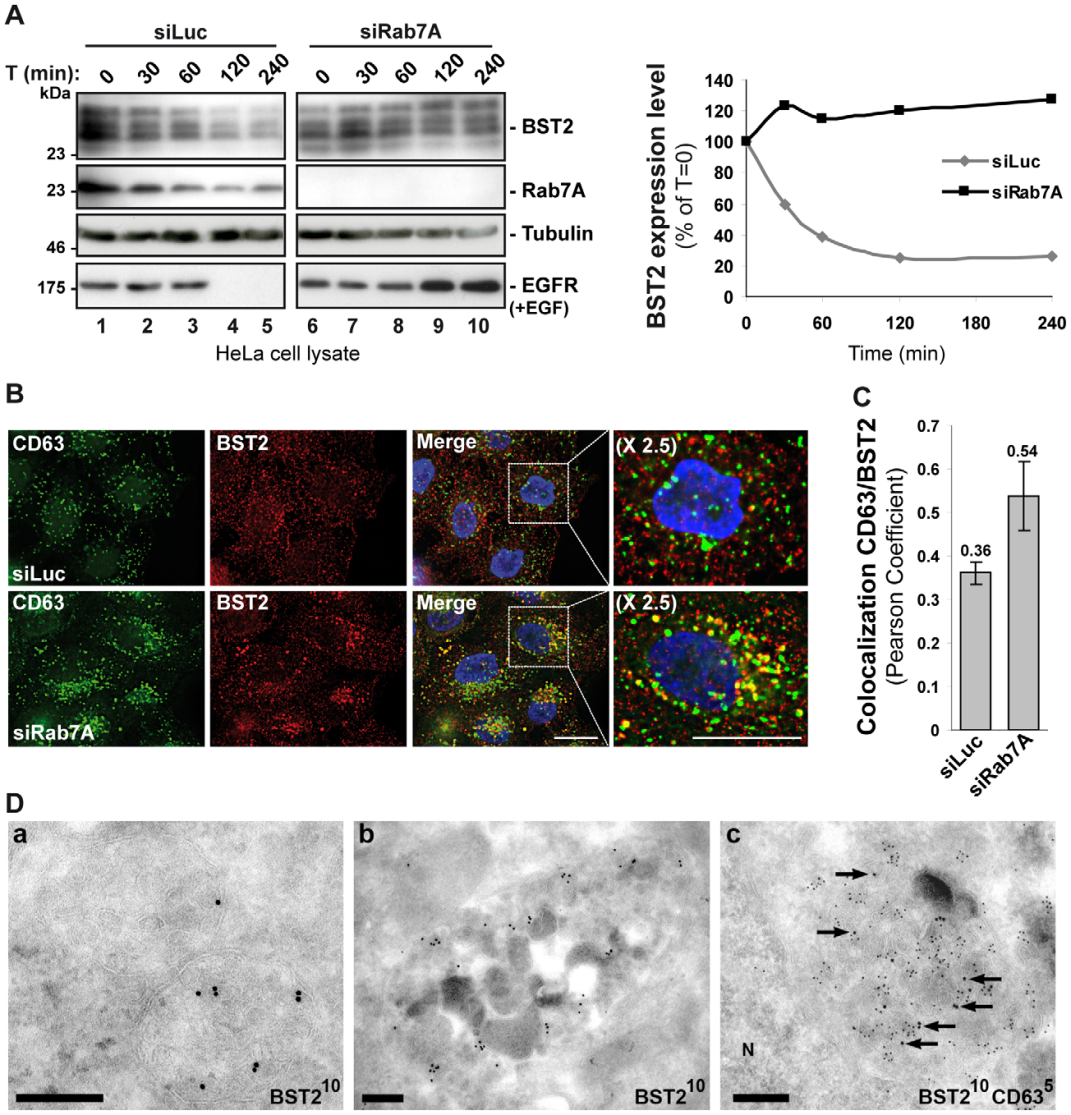
Rab7A sorts BST2 for degradation. (**A**) Analysis of BST2 turnover upon Rab7A depletion. HeLa cells transfected with either siRNA control Luciferase (siLuc) or siRNA targeting Rab7A were incubated with cycloheximide (10 µg/ml) and, where indicated, with EGF for the times indicated above each lane. Cells were lysed and equivalent amounts of each sample (50 µg of protein) were analyzed using western blotting with antibodies against BST2, Rab7A, EGF-Receptor (EGF-R) and tubulin as a loading control. For each sample, the intensity of the BST2 signal was quantified using ImageJ software and used to calculate the amount of BST2 remaining at each time point, relative to t = 0, set to 100% in the graph shown on the right. (**B**) Effect of Rab7A silencing on BST2 distribution. HeLa cells transfected with either siRNA control Luciferase (siLuc) or siRNA targeting Rab7A were permeabilized before fixation and staining with mouse polyclonal anti-BST2 and appropriate secondary fluorophore-conjugated antibodies. Cells were then labelled with mouse CD63-FITC. Scale bars = 20 µm. (**C**) CD63-BST2 colocalization was assessed by calculating the Pearson correlation coefficient on twenty images per condition using the JACoP plugin on ImageJ. (**D**) BST2 accumulates in MVB in Rab7A knockdown cells. Ultrathin cryosections of HeLa cells transfected with siRNA targeting Rab7A and infected with VSV-G-pseudotyped HIV-1 were stained with mouse (panels a, b) or rabbit (panel c) polyclonal anti-BST2 antibodies and 10 nm PAG. In panel c, sections were co-stained for CD63 with 5 nm PAG. Arrows mark some of the 10 nm PAG particles. N, nucleus. Scale bars = 200 nm.

We have previously shown that BST2 undergoes constitutive ESCRT-dependent sorting for lysosomal degradation and that this degradation is enhanced by Vpu expression [30]. As Rab7A is a key regulator of late endocytic compartments and is involved in targeting cargoes to the lysosomal degradation pathway [35]–[41], we tested whether Rab7A regulates the degradation of BST2. Uninfected HeLa cells were transfected with siRNA targeting Rab7A or a control siRNA, and the turnover of BST2 was monitored after treating the cells with cycloheximide. We observed that almost 80% of BST2 was degraded over 4 h in cells transfected with control siRNA (Figure 6A, siLuc). By contrast, the half-life of BST2 was increased in Rab7A-depleted cells (Figure 6A, siRab7A), with less than 5% of BST2 degraded after 4 h cycloheximide treatment. As an internal control, we monitored the turnover of EGF receptors (EGF-R) following EGF stimulation. Upon ligand binding, EGF-R is targeted to MVBs prior to lysosomal degradation [41], [58]–[60]. Consistent with previous studies [60], knockdown of Rab7A inhibited EGF-induced EGF-R degradation compared to control cells (Figure 6A).

We also explored the intracellular location of BST2 in the absence of Rab7A expression. Immunofluorescence analysis revealed an accumulation of BST2 in enlarged CD63+ late endocytic compartments compared to the control cells, consistent with a stabilization of the protein (Figure 6B). Quantification of the colocalization between BST2 and CD63 using Pearson’s correlation coefficient [61] validated that BST2 is retained in CD63 positive compartments after treatment with Rab7A siRNA (0.36 for control siRNA treated cells *vs* 0.54 for Rab7A siRNA treated cells; Figure 6C). Immunolabelling of ultrathin cryosections demonstrated that BST2 was present in MVB (Figure 6D) which co-stained for CD63 (Figure 6D, panel c).

To confirm these data, we analyzed the effect of overexpressing an inactive form of Rab7A, Rab7A T22N, on BST2 turnover and localization. HeLa cells were transfected with GFP-Rab7A WT or T22N. The turnover of BST2 was monitored after incubating the cells in growth medium containing cycloheximide. We noted that lower levels of BST2 were detected in cells transfected with GFP-Rab7A WT after 4 h cycloheximide treatment whereas BST2 was stabilized in GFP-Rab7A T22N expressing cells (Figure S5A, GFP-Rab7A T22N). Moreover, analysis of the distribution of BST2 in GFP-Rab7A T22N expressing cells showed a redistribution and accumulation of BST2 in intracellular compartments (Figure S5B). Together, these data indicate that the inactive form of Rab7A blocked the degradation of BST2, and show that Rab7A is involved in the constitutive turnover of BST2.

### Rab7A depletion perturbs Vpu-induced BST2 degradation

**Figure 7:**
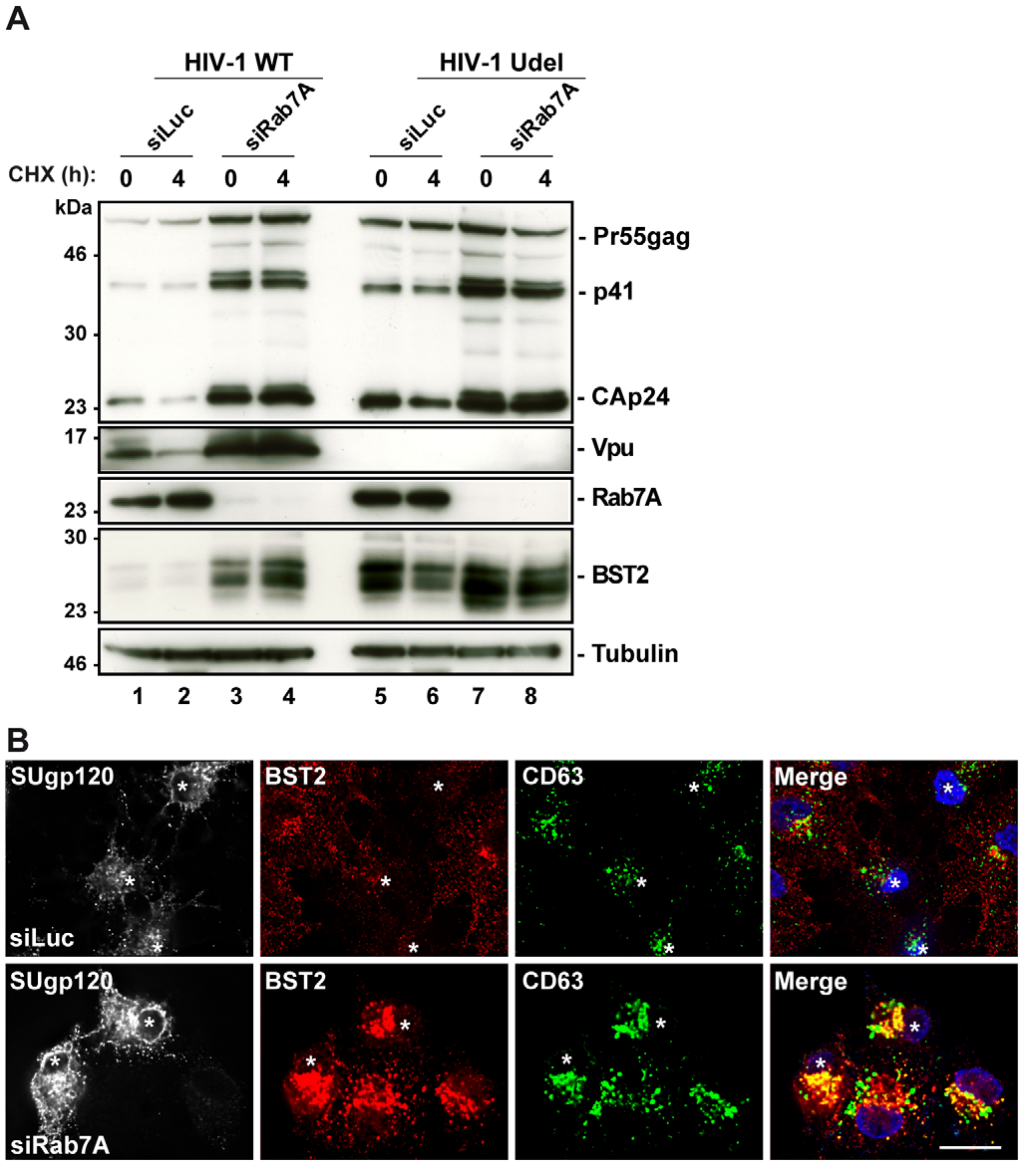
Depletion of Rab7A increases the cellular BST2 levels in the presence or absence of Vpu. (**A**) Analysis of BST2 turnover upon Rab7A depletion in HIV-1 infected cells. HeLa cells transfected with either control siRNA (siLuc) or siRNA targeting Rab7A were infected with either VSV-G pseudotyped wt NL4-3 (HIV-1 WT) or VSV-G pseudotyped Vpu-defective NL4-3 (HIV-1 Udel) HIV-1 at a MOI of 1. Forty-eight hours after infection, some of the cells were incubated with cycloheximide for 4 h and lysed. Cell lysates were analyzed by western blot. Tubulin is the loading control. These data are representative of 2 independent experiments. (**B**) Effect of Rab7A silencing on BST2 distribution in HIV-1 infected cells. HeLa cells transfected with either control siRNA (siLuc) or siRNA targeting Rab7A were infected with VSV-G pseudotyped NL4-3 HIV-1 WT and processed for immunolabelling with mouse polyclonal BST2, human HIV-1 Env (2G12) and mouse CD63-FITC antibodies. Cells were imaged by confocal microscopy. Env staining discriminates between infected cells (stars) and non-infected cells. Scale Bars = 20 µm.

It has been reported that Vpu increases the rate of BST2 degradation [30]. We thus investigated whether Rab7A participates in Vpu-induced BST2 turnover. Cycloheximide chase experiments were performed as described above on cells treated with control (siLuc) or Rab7A siRNA and infected with WT (HIV-1 WT) or Vpu-deleted (HIV-1 Udel) NL4-3 HIV pseudotyped with VSV-G (MOI  = 0.5) (Figure 7A). As previously shown, expression of Vpu enhanced BST2 degradation in infected cells. Low levels of BST2 were detected in control cells infected with HIV-1 WT compared to control cells infected with Vpu-deleted HIV-1 (HIV-1 Udel) (Figure 7A, compare lanes 1–2 with lanes 5–6). Interestingly, Rab7A knockdown stabilized BST2 over 4 hours in cells infected with HIV-1 WT (lanes 3–4) to a level similar to that observed in cells infected with HIV-1 Udel (lanes 5–6), indicating that Vpu accelerates the intracellular degradation of BST2 by a mechanism involving Rab7A. Finally, we explored the localization of BST2 in infected cells after knockdown of Rab7A. As shown in non-infected cells (Figure 6B), upon Rab7A depletion, BST2 accumulated in intracellular compartments, many of which co-stained for CD63 (Figure 7B).

Altogether, these data support the hypothesis that the late endocytic pathway, regulated by Rab7A, is involved in Vpu-induced degradation of BST2.

### Rab7A depletion reduces the cell surface expression of BST2

**Figure 8:**
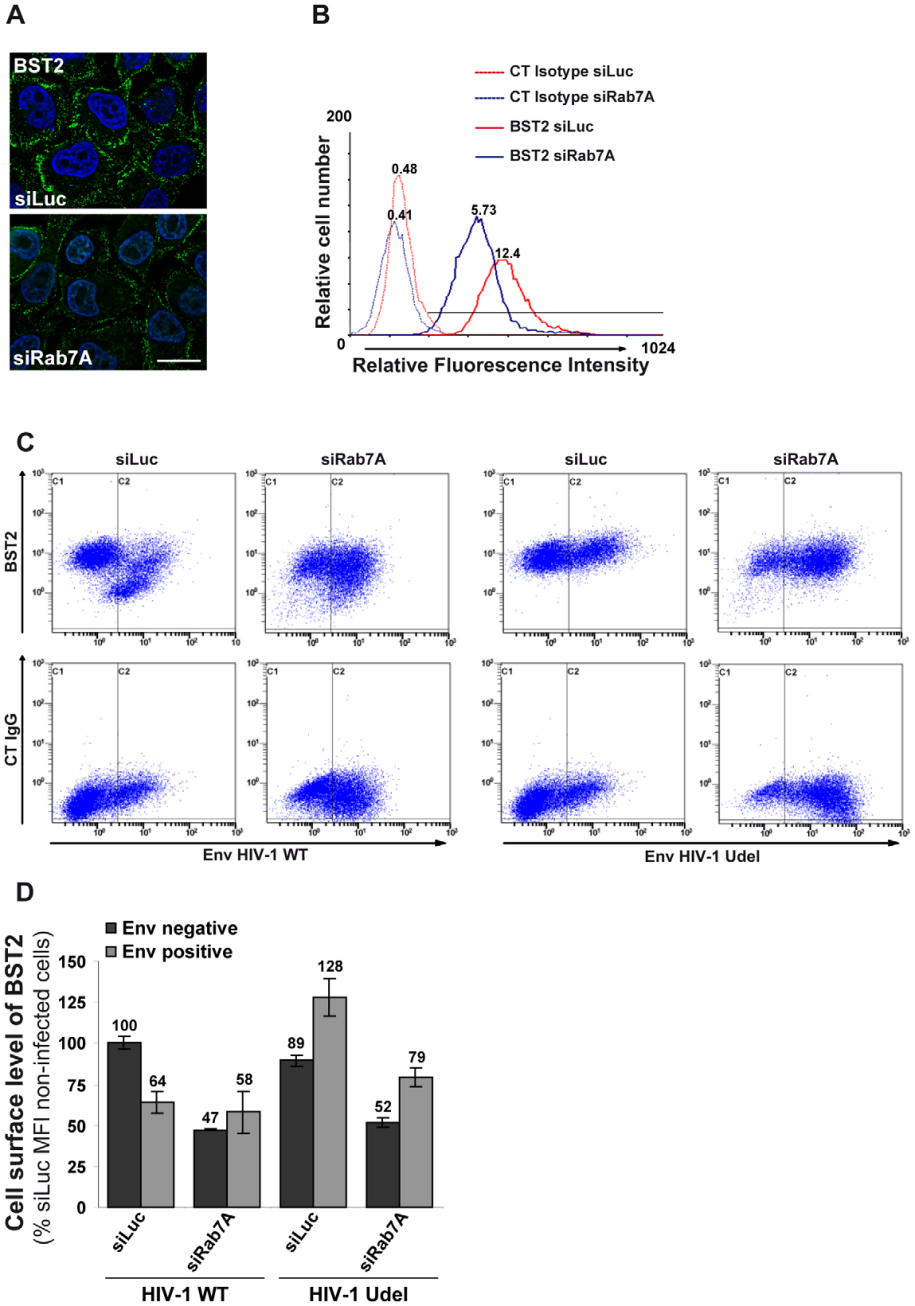
Rab7A decreases BST2 cell surface expression and is required for Vpu-induced BST2 cell surface downregulation. (**A**–**B**) Rab7A decreases BST2 cell surface expression. HeLa cells were transfected with either siRNA control Luciferase (siLuc) or siRNA targeting Rab7A (siRab7A). (**A)** Forty-eight hours after transfection, cells were stained at 4°C with anti-BST2 antibody and appropriate fluorophore-conjugated secondary antibody. Cells were then fixed with PFA. Scale bars = 20 µm. (**B**) Forty-eight hours after transfection, cells were stained with a mouse monoclonal antibody against BST2 or isotype control mouse IgG1. Cells were then stained with Alexa 647-conjugated donkey anti-mouse antibody, fixed and processed for flow cytometry analysis. These data are representative of 3 independent experiments. (**C**–**D**) Rab7A is required for Vpu-induced cell surface downregulation of BST2. HeLa cells transfected with either control siRNA (siLuc) or siRNA targeting Rab7A were infected with VSV-G pseudotyped NL4-3 HIV-1 (WT or Udel) at a MOI of 0.5. Twenty-four hours later the cells were stained with mouse monoclonal antibody against BST2 (BST2; upper panels) or control mouse IgG1 as an isotype control (CT IgG; lower panels), along with human anti-Env antibody (2G12). The cells were then stained with Alexa 647-conjugated donkey anti-mouse and PE-conjugated donkey anti-human antibodies, fixed and processed for flow cytometry analysis. (**C**) Dot plot. Vertical lines indicate the gates set using non-infected cells stained as indicated. Gate C1 represents non-infected cells and gate C2 infected cells. These data are representative of 3 independent experiments. (**D**) Bar graph representation of the cell surface levels of BST2 (Mean of fluorescence Intensity; MFI) on Env negative cells (black bars) and Env positive cells (grey bars) for each siRNA condition. Bars represent the means of the MFI values ± SD from 3 independent experiments.

We analyzed whether the intracellular accumulation of BST2 observed upon Rab7A depletion affected the cell surface expression of BST2. Uninfected HeLa cells were transfected with control siRNA or with siRNA targeting Rab7A and the cell surface levels of BST2 were assessed by immunofluorescence staining and flow cytometry (Figures 8A and 8B). In Rab7A-depleted cells, a marked decrease of cell surface BST2 was observed by immunofluorescence (Figure 8A) and flow cytometry (Figure 8B), compared to cells transfected with control siRNA (siLuc), while a higher expression of CD63 and EGF-R was detected at the cell surface (data not shown). Together, our data suggest that depletion of Rab7A slows the degradation of BST2 and induces its accumulation in intracellular compartments, with a concomitant reduction of BST2 expression at the cell surface, probably due to a block in the recycling of BST2 from endosomes.

We also explored the effect of Rab7A silencing on Vpu-mediated downregulation of cell surface BST2. siRNA transfected HeLa cells were infected with VSV-G pseudotyped WT or Udel NL4-3 HIV-1 at a MOI of 0.5 so that approximately 50% of the cells were infected. Cell surface levels of BST2 were assessed by flow cytometry (Figures 8C and 8D). Cell surface staining of Env (SUgp120) was used to distinguish non-infected cells (Figures 8C and 8D, gate C1 and black bars) from infected cells (Figures 8C and 8D, gate C2 and grey bars). In control cells (siRNA Luc) infected with HIV-1 WT, BST2 cell surface expression decreased by ≥35% compared with non-infected cells, consistent with previous studies [28], [29], [54], [56], [62]. Depletion of Rab7A reduced cell surface BST2 levels on non-infected cells (≥35%) or on Udel infected cells (≥38%), compared to the corresponding control cells (siLuc). Moreover, cell surface expression of BST2 was not downregulated further upon HIV-1 infection in Rab7A knockdown cells (Figure 8C, compare gate C2 to C1 on the upper 2nd panel).

### A fraction of BST2 persists at the cell surface upon Rab7A depletion

BST2/Tetherin impedes the release of HIV-1 by physically tethering fully formed mature particles to the plasma membrane of infected cells. Vpu abrogates this function by reducing the cell surface expression of BST2, thus allowing efficient virus release [28], [29], [52], [54], [55]. Our data show that Rab7A depletion impairs the release of HIV (Figures 2 and 3), an effect that seems to be related to the expression of BST2 (Figure 5). However, surprisingly, the cell surface expression of BST2 was reduced in the Rab7A knockdown cells (Figure 8).

**Figure 9:**
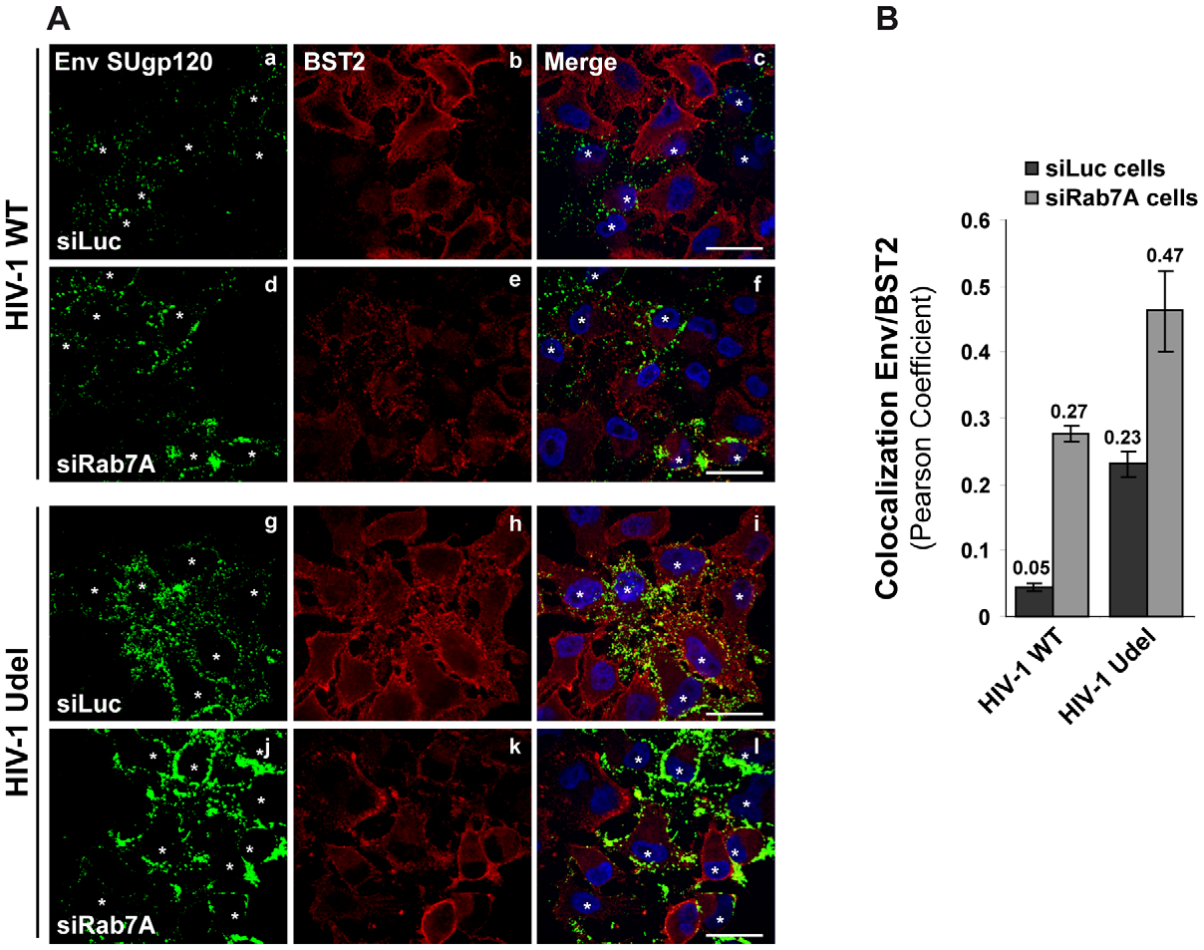
Env SUgp120 and BST2 proteins colocalize at the cell surface of Rab7A-depleted cells. HeLa cells transfected with either siRNA control Luciferase (siLuc) or siRNA targeting Rab7A (siRab7A) were infected with either VSV-G pseudotyped wt NL4-3 HIV-1 (HIV-1 WT) viruses or VSV-G pseudotyped Vpu-defective NL4-3 (HIV-1 Udel) viruses. (**A**) Twenty-four hours after infection, cells were stained at 4°C with anti-Env and anti-BST2 antibodies and appropriate fluorophore-conjugated secondary antibodies. Cells were then fixed with PFA, permeabilized and labelled with DAPI. Infected cells are indicated with white stars. Scale bars = 20 µm. (**B**) Env-BST2 colocalization was assessed by calculating the Pearson correlation coefficient on twenty images per condition using the JACoP plugin on ImageJ.

To understand how Rab7A depletion can lead to reduced HIV-1 release and to the accumulation of viruses at the cell surface (Figure 4), despite reducing cell surface BST2 levels, we analyzed more closely the location of cell surface BST2 in infected siRNA treated cells by immunofluorescence staining (Figure 9). HeLa cells were treated with siRNA against Rab7A (siRab7A) or control siRNA (siLuc), then infected with VSV-G pseudotyped wt and Udel viruses and processed for labelling of cell surface Env and BST2. Infected cells, identified by staining for Env, are indicated on Figure 9A with white stars. Control cells (siLuc) infected with wt HIV-1 showed reduced BST2 staining at the cell surface compared to neighbouring non-infected cells (Figure 9A, HIV-1 WT, panels b and c). As expected, the cell surface expression of BST2 was not changed in control cells (siLuc) infected with Udel HIV-1 (Figure 9A, HIV-1 Udel, panels h and i). These results are consistent with Vpu-induced BST2 cell surface downregulation [29], [54], [56], [62]. Similar to the results shown in Figure 8, silencing of Rab7A induced an overall decrease of BST2 cell surface expression in both non-infected and infected cells (Figure 9A, compare panels b and h to e and k, respectively). Quantification of the colocalization between BST2 and Env at the cell surface using Pearson’s correlation coefficient [61] revealed the presence of BST2 in Env positive areas in Rab7A-depleted cells to a similar extent as in control cells infected with Udel viruses (Figure 9B), whereas almost no colocalization was observed between BST2 and Env in control cells infected with wt viruses. Similar results were obtained after quantification of MAp17/BST2 colocalization at the cell surface (Figure S6). These data suggest that, upon depletion of Rab7A, a fraction of BST2 remains in Env and MA positive patches at the cell surface, even in the presence of Vpu.

## Discussion

The small GTPase Rab7A plays a major role in regulating trafficking through the late endocytic pathway. Indeed, Rab7A controls the transport of cargoes through late endosomes, facilitates endosome maturation and regulates the interaction of endosomes with the cytoskeleton [35]–[46]. Here we show that Rab7A expression is required for the efficient propagation of infectious HIV-1. Several components of the cellular trafficking machineries have previously been shown to be involved in the late steps of the HIV-1 replication cycle. These include the ESCRT machinery, as HIV-1 Gag recruits TSG101, an ESCRT-I component, or ALIX, an ESCRT-associated protein, to mediate the final scission events that release virus particles from the plasma membrane [24]–[27]. Moreover, HRS, a component of the ESCRT-0 complex, is involved in Vpu-induced BST2 cell surface downregulation and degradation, thereby contributing to efficient HIV-1 release [30]. The clathrin adaptor complexes AP-1 and AP-3, as well as TIP47, ARF and GGA proteins, also contribute to Gag and Env trafficking through the cell and are required for efficient production of infectious HIV-1 [9]–[20]. The involvement of Rab7A in the release of infectious HIV-1 further highlights the importance of the vesicular trafficking machinery in the life cycle of HIV-1.

A number of Rab proteins have previously been implicated in HIV-1 replication. Rab5A, which coordinates endocytosis and early endosome function, has been reported to regulate the early events of HIV-1 infection in polarized human trophoblasts [63]. Silencing of Rab5A did not significantly alter HIV-1 propagation in our assay (Figure 1), possibly because HIV entry in HeLa P4-R5 cells may occur mainly by fusion of the virus with the cell surface, a Rab5A-independent process. A functional genomic screen using siRNAs identified the Golgi-localized Rab6A, which controls retrograde transport from the Golgi apparatus to the endoplasmic reticulum (ER), as a cellular factor required for HIV-1 dissemination [49] and efficient HIV entry. The late steps of HIV-1 replication, including trafficking of Gag and Env and the assembly and release of new viral particles, also depend on the expression of key Rab proteins. Efficient HIV-1 replication and Gag trafficking requires expression of Rab9A [48], a Rab protein regulating sorting of cargoes along the late endocytic pathway from late endosomes to the TGN. Our data corroborate these findings, showing that Rab9A expression is important for efficient spreading of HIV-1, and also illustrate that Rab proteins regulating exocytic membrane traffic support efficient HIV propagation in cell culture (Figure 1). We observed that Rab4A, Rab8A and Rab11A, key regulators of the recycling pathway, are required for HIV dissemination in our assay, in part confirming previous reports that Rab11A is implicated in HIV replication [48]. The precise steps that are regulated by Rab4A and Rab8A are not defined, and further studies will be necessary to characterize the role of these two Rab proteins in HIV-1 replication.

The present study highlights an unsuspected and major role for Rab7A, a key player of the late endocytic pathway, in the HIV replication cycle. Rab7A depletion strongly decreased HIV-1 dissemination in HeLa P4-R5 cells (Figure 1). We observed a significant, though mild effect of Rab7A knockdown on HIV-1 entry. However, this only accounts for a minor part of the dramatic alteration of HIV propagation observed in these cells (Figure S2). Indeed, our virus release assays documented that Rab7A knockdown considerably reduced HIV-1 infectivity and release (Figures 2 and 3). Analysis of viruses produced by Rab7A knockdown cells suggested that Rab7A is required for efficient processing of the Env glycoprotein; virus particles produced in Rab7A-depleted cells incorporated mainly the immature Env precursor, gp160, and were poorly infectious (Figures 3B and 3C). Moreover, our analysis of Env processing revealed a defect in Env maturation in the absence of Rab7A expression (Figures 3D and 3E). Env maturation to the TMgp41 and SUgp120 subunits by cellular endoproteases is essential for HIV-1 infectivity [64], [65]. Rab7A may regulate the trafficking of Env and/or furin or furin-like proteases that are responsible for Env processing. In the absence of Rab7A, trafficking of Env through furin-containing compartments may be reduced, leading to an accumulation of Env precursors in the producer cells and on viruses. Rab7A knockdown could also induce mislocalization of Env and/or furin, reducing their encounter in the cell and thus Env processing. The Env maturation defect could also be the consequence of the increased levels of Env in Rab7A knockdown cells. Alteration of the late endocytic pathway upon Rab7A depletion could lead to an accumulation of Env in endocytic compartments or the TGN to levels that exceed the processing capacity of the cellular endoproteases, thereby interfering with the maturation of newly synthesized Env.

In addition to the effect on Env processing and HIV-1 infectivity, Rab7A silencing impaired HIV-1 release from infected cells (Figure 2). Fewer virus particles were released into the medium upon Rab7A depletion, and instead viruses accumulated in large clusters at the cell surface, as demonstrated by immunofluorescence and immuno-electron microscopy experiments (Figure 4). In addition, we observed an increase in the levels of cell-associated Env and Gag proteins. As Rab7A is a key regulator of the late endocytic pathway, which has been previously described to be involved in the degradation of excess viral proteins in infected cells [50], [51], alterations in endo/lysosomal degradation in Rab7A-depleted cells could be the primary cause of the buildup of viral proteins. In addition, the accumulation of clusters of viruses at the surface of infected Rab7A knockdown cells (Figure 4 and S4) may also contribute to the increased levels of viral products associated with the cells. The accumulation of virus clusters at the cell surface has been previously observed following infection of BST2-expressing cells with Vpu-defective HIV-1, suggesting that the effect of Rab7A knockdown on HIV release could be related to BST2 expression [28], [29], [57]. This hypothesis is supported by the fact that HIV release was less affected when Rab7A was silenced in cells devoid of BST2 (e.g. in non-restrictive 293T cells, Figures 5A and 5B) or in BST2-depleted HeLa cells (Figures 5C and 5D).

BST2/Tetherin has been identified as a cellular restriction factor that impedes the release of HIV-1 by physically tethering fully formed mature virus particles to the plasma membrane of infected cells [28], [29], [55]. The HIV-1 protein Vpu acts as a countermeasure to BST2, downregulating BST2 from the cell surface and enhancing its degradation, thereby rescuing efficient virus release [28], [29], [54], [55]. BST2 has been shown to internalize *via* clathrin-coated pits and recycle from endosomes, probably *via* the trans-Golgi network (TGN) and/or recycling endosomes [62], [66], while a proportion of the protein is degraded in lysosomes [30]. In HIV infected cells, Vpu counteracts the restriction imposed by BST2 by inhibiting this cycling and enhancing the lysosomal degradation of BST2 [30], [54], [56], [67], thereby decreasing the levels of cellular and cell surface BST2 [28], [29], [52]. We show here that Rab7A, which regulates exit from late endosomes/MVB, is required for sorting of BST2 to lysosomes and degradation. Depletion of Rab7A by RNA interference prevents lysosomal degradation of the protein, thereby increasing cellular BST2 levels (Figure 6), both in the presence and absence of Vpu (Figure 7). Rab7A knockdown may also block exit from MVB to other routes. As a result, we observed a large build-up of BST2 in late endosomes and MVB/CD63+ compartments (Figures 6B and 6D). Recycling of BST-2 to the cell surface might also be reduced, leading to lower BST2 expression at the cell surface (Figure 8). In addition reduced access of newly synthesized BST2 to the cell surface could also contribute to the decreased cell surface expression of BST2 observed upon Rab7A depletion. Alternatively Rab7A knock-down may induce an alteration of the expression level or localization of a cellular cofactor regulating BST2 trafficking. Nonetheless, some newly synthesized BST2, together with some leak-through recycling of BST2 from MVB, could still reach the cell surface (Figure 9), leading to the tethering of virus to the plasma membrane (Figure 4).

Exactly how Rab7A supports the sorting of BST2 to lysosomes and its degradation is not clear. BST2 was recently shown to be ubiquitinated and Vpu expression was shown to increase this ubiquitination [68], [69]. Furthermore, β-TRCP2, a subunit of the Skp1-cullin1-F-Box (SCF) ubiquitin ligase complex is required for Vpu to downregulate BST2 expression [53], [54], [56], [62] and the SCF ubiquitin ligase complex was shown to be recruited to endocytic membranes [70]. We might speculate that Rab7A knockdown alters β-TRCP2 recruitment, perturbing Vpu-induced ubiquitination of BST2, and thus blocking its downregulation. We cannot exclude that other E3 ligases or unidentified cellular cofactors of BST2 or Vpu that are important for Vpu-induced downregulation of BST2 could be mislocalized, downregulated or upregulated upon Rab7A knockdown. It has recently been shown that RING-type E3 ubiquitin ligase, BCA2/Rabring7 (Breast cancer-associated gene 2), a cellular cofactor of BST2 contributes to the restriction of HIV-1 particle production by accelerating the internalization and degradation of viral particles [71]. BCA2 directly binds Rab7A and thereby plays a crucial role in vesicle trafficking to late endosomes and lysosomes [72], [73]. Silencing of Rab7A may alter this function of BCA2. However, overexpression of BCA2 in BST2 restrictive cells did not lead to the same phenotype as that observed on HIV production in Rab7A-depleted cells, suggesting that the role of Rab7A on HIV release is not related to BCA2. However, these differences could be explained by the fact that overexpression of this Rab7 effector does not impair the late endocytic pathway, but instead activate it [71]. Further experiments will be necessary to decipher the molecular mechanisms by which Rab7A blocks Vpu-induced downregulation of BST2. Indeed, Rab7A could also regulate another tethering factor that may contribute to retaining viruses. This idea is supported by the observation that, after double knockdown of both Rab7A and BST2 (Figures 5C and 5D), HIV release was still impeded, though not as much as in BST2-expressing cells.

Rab7A has been previously shown to be involved in the entry of several viruses such as Semliki Forest virus, Parvoviruses, Dengue and West Nile virus, by sorting them to late endosomes after their internalization [74]–[77]. Here, we show that Rab7A regulates the production of infectious HIV-1 particles. Rab7A is required for efficient Env processing, allowing the proper incorporation of mature Env glycoproteins. Rab7A also promotes HIV release by controlling the trafficking and degradation of BST2, and by contributing to the mechanism by which Vpu counteracts this restriction factor. These studies highlight new roles of a major component of the late endocytic pathway, Rab7A, in the late steps of the HIV replication cycle. Although the precise molecular and cellular mechanisms by which Rab7A mediates these effects remain to be elucidated, our data bring significant new insights to understanding HIV assembly, restriction by BST2/Tetherin and how Vpu uses cellular machineries, and particularly the endocytic pathway, to counteract this host cell restriction factor, thereby favoring HIV-1 dissemination.

## Materials and Methods

### Cell culture

HeLa, HeLa P4-R5 MAGI (NIH AIDS Research and Reference Reagent Program, Division of AIDS, NIAID), HeLa TZM-bl (NIH AIDS Research and Reference Reagent Program, Division of AIDS, NIAID) and HEK 293T cells were grown in DMEM plus glutamine, antibiotics and 10% decomplemented-FCS (fetal calf serum) (GibcoBRL, Invitrogen, France). HeLa P4-R5 MAGI cell cultures were supplemented with 100 µg/mL geneticin and 1 µg/mL puromycin.

### siRNA transfection and reagents

Small interfering RNA (siRNA) transfections were performed with 5 to 30 nM siRNA using Lipofectamine RNAiMAX (Invitrogen), according to the reverse transfection procedure described in the manufacturer’s instructions.

ON-TARGETplus SMARTpool Rab1A (L-008283-00-0005), Rab4A (L-008539-00-0005), Rab5A (L-004009-00-0005), Rab6A (L-008975-00-0005), Rab8A (L-003905-00-0005) and Rab11A (L-004726-00-0005), synthesized by Thermo Scientific Dharmacon (Perbio Science, France), target Rab1A, Rab4A, Rab5A, Rab6A, Rab8A, and Rab11A mRNAs, respectively (see also Figure S1). Rab7A siRNA (5’GGGAGAUUCUGGAGUCGGGAAdTdT3’) and Rab7A-2 siRNA (5’CCACAAUAGGAGCUGACUUdTdT3’) target the Rab7A mRNA at positions 274–294 and 348–366 respectively. Rab9A siRNA (5’CGGCAGGUGUCUACAGAAGdTdT3’) targets the Rab9A mRNA at positions 573–591. The On-TARGETplus SMART pool siRNA targeting BST2 was purchased from Dharmacon (L-011817-00). The siRNA against luciferase (5’CGUACGCGGAAUACUUCGAdTdT3’) which targets the *P. Pyralis* firefly GL2 luciferase mRNA sequence (positions 515–535) was used as control (Thermo Scientific Dharmacon).

### Viral stocks

Stocks of wild type NL4-3 HIV-1 (NIH AIDS Research and Reference Reagent Program, Division of AIDS, NIAID) or NL4-3/Udel HIV-1 (from Dr. K. Strebel [78]) were obtained by transfection of HEK 293T cells (2×10^6^) with 6 µg of the corresponding proviral DNA, using the Fugene reagent as recommended by the manufacturer (Roche Diagnostics, France). VSV-G pseudotyped virus was generated by transfection of HEK 293T cells (2×10^6^) with 1.5 µg of VSV-G expression vector (pMD.G) along with 4.5 µg of HIV-1 proviral DNA (pNL4–3, pNL4-3/Udel). Supernatants of the transfected cells were collected after 48 h, filtered and quantified for the HIV-1 CAp24 antigen by ELISA (Innotests HIV Antigen mAb, Innogenetics, France). Viral titres were assessed by infection of the indicator cells HeLa TZM-bl (bearing the β-galactosidase gene under the control of HIV-1 LTR) with serial dilutions of the stocks, followed by a β-galactosidase coloration of the cells and counting of blue cells.

### Recombinant DNA and transfection procedures

Rab7A WT cDNA was cloned into pEGFP-C vector (Clontech, France). The inactive form of Rab7A (Rab7A T22N) was made by PCR mutagenesis using the QuikChange II site directed mutagenesis kit (Stratagene, France). All mutagenesis and subclonings were verified by DNA sequencing. Transient transfections of HeLa cells (5×10^5^) with mammalian expression vectors (3 µg) were performed using Lipofectamine LTX with PLUS Reagent (Invitrogen), following the manufacturer's instructions.

### HIV-1 propagation assay

HeLa P4-R5 MAGI cells (1×10^5^) transfected with siRNA were infected with NL4-3 HIV-1 at a MOI of 0.005. Six hours after infection, cells were washed and placed in fresh medium. Supernatants were collected every 24 h for 4 days. HIV-1 propagation was monitored by the X-gal staining of infected cells and by the ELISA quantification of CAp24 antigen released into the cell culture supernatants after multiple rounds of infection. For X-gal staining, cells were fixed with 0.5% glutaraldehyde solution and stained with X-Gal solution (4 mM potassium ferrocyanide hydrate, 4 mM potassium ferricyanide, 2 nM MgCl_2_, and 0.4 mg/ml X-Gal, 5-bromo-4-chloro-3-indolyl β-D-galactopyranoside).

In parallel, infected cell cultures were lysed and extracts were analyzed for Rab protein expression by western blotting using rabbit anti-Rab1A (Abcam, UK), goat anti-Rab4A (Santa Cruz Biotechnology, France), rabbit anti-Rab5A (Santa Cruz Biotechnology), rabbit anti-Rab6A (Santa Cruz Biotechnology), mouse anti-Rab7A (Sigma), goat anti-Rab8A (Santa Cruz Biotechnology), mouse anti-Rab9A (ABR, France), rabbit anti-Rab11A (Santa Cruz Biotechnology), and mouse anti-tubulin (DM1A, Sigma) antibodies.

### HIV-1 entry assay

For the entry assays, CD4^+^ CCR5^+^ CXCR4^+^ HeLa P4-R5 MAGI cells (5×10^5^) transfected with siRNA were infected with wild-type NL4-3 HIV-1 or wild-type NL4-3 HIV-1 pseudotyped with VSV-G at a MOI of 0.005. Cells were washed 4 h later and placed in fresh medium supplemented with AMD3100 (Sigma), a CXCR4 inhibitor. After 24 h, cells were fixed with 0.5% glutaraldehyde solution and stained with X-Gal solution as described above.

### HIV-1 production and infectivity assays

For HIV-1 production assays in a single round of infection, HeLa cells (2×10^5^) were treated with siRNA as described above. HEK 293T cells (3×10^5^) were treated with 20 nM siRNA. After 48 h, the cells were infected with NL4-3 (WT or Udel) HIV-1 pseudotyped with VSV-G for 6 h at a MOI of 0.5. Twenty-four hours after infection, supernatants were collected, 0.45 µm-filtered and used for HIV-1 CAp24 quantification by ELISA (released CAp24). Viral particles released into the supernatant were pelleted through a 20% sucrose cushion by ultracentrifugation at 150,000 g for 90 min and resuspended in Laemmli sample buffer. Pelleted viruses were analyzed by western blotting using rabbit anti-CAp24 (4250; NIH AIDS Research and Reference Reagent Program, Division of AIDS, NIAID). The cell lysates were analyzed by western blotting using rabbit anti-CAp24, rabbit anti-Vpu (NIH AIDS Research and Reference Reagent Program, Division of AIDS, NIAID), mouse anti-Rab7A, rabbit anti-BST2 (NIH AIDS Research and Reference Reagent Program, Division of AIDS, NIAID) and mouse anti-Tubulin (Sigma) antibodies.

In a single round infection assay, the titres of released viruses were determined by infection of the indicator cells HeLa TZM-bl. 24 hours later, cells were fixed with 0.5% glutaraldehyde and stained with X-Gal solution (see above). HIV-1 infectivity corresponded to the ratio of the titre of the produced virus to the quantity of CAp24 detected in the supernatant.

### Incorporation assays and quantification of HIV-1 viral genomic RNA

Forty-eight hours after siRNA transfection, HeLa cells were infected with VSV-G pseudotyped wt NL4-3 HIV-1 (NL4-3 WT) treated with DNase I (Roche Diagnostics, 0.1 units/µl) plus MgCl_2_ (10 mM) at 37°C for 1 h to eliminate residual proviral DNA used to prepare viral stocks. Supernatants of infected cells were harvested 24 h later, 0.45 µm-filtered, and virions pelleted through a 20% sucrose cushion by ultracentrifugation at 150 000 g for 90 min, and resuspended in PBS. Producer cells were collected and pelleted. Half of the producer cells and pelleted virus preparations were lysed in lysis buffer (50 mM Tris pH 7.5, 150 mM NaCl, 2 mM EDTA, 1% (v/v) Triton X-100, 0.1% (v/v) sodium deoxycholate) and quantified by CAp24 ELISA, or analyzed by western blotting using rabbit anti-CAp24, mouse anti-SUgp120 (110H, Hybridolab, France) and human anti-TMgp41 (41A, Hybridolab), rabbit anti-MAp17 (VU47; NIH AIDS Research and Reference Reagent Program, Division of AIDS, NIAID), mouse anti-Rab7A, and mouse anti-Tubulin antibodies.

The remaining half of the producer cell and virus preparations was used for quantification of viral genomic RNA. Prior to RNA purification, non-infected HeLa cells (1×10^6^) were added to each virus sample to control for the efficiency of RNA extraction and reverse transcription assays of the viral preparation for quantitative real-time PCR as described below. RNA from the virus samples or producer cells was purified (RNeasy Mini Kit, Qiagen, France) and treated with DNase I (0.5 U/µl) for 20 min at 30°C. DNase I was then heat-inactivated for 10 min at 75°C. 1 µg of total RNA was used for reverse transcription by using random hexamers with M-MLV reverse transcriptase (Moloney Murine Leukemia Virus RT; Invitrogen) to generate cDNA for real-time PCR analysis. To remove RNA complementary to the cDNA, samples were treated for 20 min at 37°C with RNAse H (0.05 U/µl; Invitrogen) and RNAse (0.005 µg/µl; Roche Diagnostics). As a negative control, an equivalent amount of RNA was used to perform the identical reaction in the absence of M-MLV RT. Real-time PCR was performed by using LightCycler FastStart DNA MasterPlus SYBR Green I reaction mix on a LightCycler instrument with LightCycler software (Version 3.5; Roche Applied Science). Real-time PCR conditions consisted of four consecutive segments as follows: a preincubation segment of 1 cycle of 10 min at 95°C; an amplification segment consisting of 40-45 cycles of 2 s at 95°C, 5 s at 55°C, and 5 s at 72°C; and finally a melting-curve segment and a cooling segment, which were performed as described by the kit's manufacturer. Unspliced genomic HIV-1 RNA (gRNA) was selectively amplified by using primers La8.1 (5’-CTGAAGCGCGCACGGCAA-3’) and L9 (5’-GACGCTCTCGCACCCATCTC-3’) [79]. This pair forms a 100 bp-amplified fragment corresponding to HIV-1 NL4-3 *gag* sequences from positions 705 to 805. To determine the relative quantity of unspliced viral transcripts for each sample, cDNA corresponding to human GAPDH was amplified (GAPDH1: 5’-GGGTCATCATCTCTGCACCT-3’ - GAPDH2: 5’-GGTCATAAGTCCCTCCACGA-3’) and used as an internal control for normalization.

### SDS-PAGE and western blot analysis

Cells were lysed for 30 min at 4°C using lysis buffer (50 mM Tris pH 7.5, 150 mM NaCl, 2 mM EDTA, 1% (v/v) Triton X-100, 0.1% (v/v) sodium deoxycholate), supplemented with complete protease inhibitor cocktail (Roche Diagnostics). Cell lysates were spun for 10 min at 20,000 g and supernatants were recovered and mixed with 2x Laemmli sample buffer, (Sigma). Samples were boiled and proteins resolved on 12% SDS–PAGE gels. Proteins were transferred onto Hydrophobic polyvinylidene difluoride (PVDF) membranes and incubated in blocking solution (TBS–0.05% (v/v) Tween 20 supplemented with 5% (w/v) milk) for 30 min. Blots were rinsed with TBS containing 0.05% (v/v) Tween 20 and incubated with primary antibody in the blocking solution overnight. The membranes were rinsed as before and incubated for 30 min with the appropriate HRP-conjugated secondary antibody diluted in the blocking solution. The membranes were rinsed again and immunodetections were performed using the enhanced chemiluminescence ECL substrate (GE healthcare Europe GMB, France).

For Env quantification, the membranes were incubated with anti-SUgp120 (110H) and TMgp41 (41A), washed and incubated with Dylight-800 Goat Anti-mouse IgG antibody (KPL, Gaithersburg, USA). The corresponding signals were then acquired with the Odyssey Infrared Imaging System (Li-Cor) for further quantification using ImageJ software.

### Immunofluorescence microscopy

For intracellular staining of CA and Env, cells were grown on cover slips, fixed with 4% (w/v) paraformaldehyde (PFA) in PBS for 20 min, permeabilized and blocked with 0.2% (w/v) BSA/0.1% (w/v) saponin in PBS (blocking solution) for 30 min. Cells were then incubated for 30 min with mouse anti-CAp24 (25A, Hybridolab) and human anti-Env HIV-1 gp120 (2G12, National Institute for Biological Standards and Control Centralised Facility for AIDS Reagents, NIBSC) in blocking solution. Cells were washed and incubated for 30 min with Alexa 488-conjugated donkey anti-mouse and Alexa 594-conjugated goat anti-human antibodies (Invitrogen).

For extracellular staining of Env, living cells were incubated for 1 h at 4°C with human anti-Env HIV-1 gp120 antibody (2G12). Cells were then labelled with Alexa 594-conjugated goat anti-human antibody, washed, fixed with 4% PFA in PBS for 20 min, permeabilized and blocked for 30 min in blocking solution. Cells were then incubated for 30 min with mouse anti-MAp17 (18A, Hybridolab) in blocking solution as described above, washed and labelled for 30 min with Alexa 488-conjugated donkey anti-mouse antibody.

For intracellular staining of BST2, cells grown on cover slips were permeabilized in PBS/0.1% BSA/0.05% saponin before fixation with 4% PFA in PBS for 15 min [80]. PFA-fixed cells were permeabilized and blocked for 30 min, and incubated for 30 min with mouse polyclonal anti-BST2 (Abnova, Germany) alone or along with anti-Env HIV-1 gp120 antibody (2G12). Cells were then washed and incubated for 30 min with appropriate fluorophore-conjugated secondary antibodies.

For colabelling of BST2 with CD63, HeLa cells transfected with either siRNA control Luciferase (siLuc) or siRNA targeting Rab7A were labelled with mouse polyclonal anti-BST2 antibody followed by an Alexa 594-conjugated donkey anti-mouse antibody. Cells were then incubated in blocking solution containing mouse serum (10%) for 20 min before immunolabelled with mouse anti-LAMP-3(CD63)-FITC (MX-49.129.5, Santa Cruz).

For extracellular staining of BST2, living cells were incubated for 1 h at 4°C with mouse monoclonal anti-BST2 (Abnova) alone or together with human anti-Env HIV-1 gp120 (2G12). Then, cells were labelled with appropriate fluorophore-conjugated secondary antibodies, washed, fixed with 4% PFA in PBS for 20 min, permeabilized and blocked for 30 min. For MAp17/BST2 colabelling, living cells were incubated for 1 h at 4°C with rabbit polyclonal anti-BST2 (NIH AIDS Research and Reference Reagent Program, Division of AIDS, NIAID), then labelled with appropriate fluorophore-conjugated secondary antibodies, washed, fixed with 4% PFA in PBS for 20 min, permeabilized and blocked for 30 min. Cells were then incubated for 30 min with mouse anti-MAp17 (18A, Hybridolab) in blocking solution as described above.

Stained cells were analyzed with a Leica DMI 6000 microscope or Leica Spinning Disk confocal microscope. Series of optical sections were recorded and image processing was performed using ImageJ and Adobe Photoshop CS2 software. Quantitative Env/BST2 and CD63/BST2 colocalization analysis was done with JACoP tool (ImageJ software) using Pearson's correlation coefficient [61]. The estimation of Pearson’s correlation coefficient is one of the standard techniques applied for matching one image to another in order to describe the degree of overlap between two channels, taking into account the environment of each pixel. A value of +1 is the result of complete co-localization between two channels. The Pearson coefficient was calculated for twenty images per condition, with about 10 cells per image. Bars represent the mean ± SD from each image.

### Immunolabelling of cryosections for electron microscopy

Cells transfected with siRNAs and infected with VSV-G pseudotyped wt NL4-3 HIV-1 were fixed in 4% PFA in 0.1 M sodium phosphate buffer, pH 7.4, embedded in gelatine, and frozen for cryosectioning, as described previously [81], [82]. For immuno-EM, ultrathin cryosections (50 nm) were quenched in 50 mM glycine-50 mM NH_4_Cl in PBS and stained with the human anti-Env mAb 2G12 and 10 nm PAG (Protein A gold reagents were obtained from the EM Lab, Utrecht University, Utrecht, The Netherlands). Sections were fixed in 1% (v/v) glutaraldehyde for 10 min, re-quenched in 50 mM glycine-50 mM NH_4_Cl in PBS and incubated with mouse antibodies against CAp24 (38:96K and EF7, ARP365 and 366, respectively, from B. Wahren, National Bacteriological Laboratory, Stockholm, Sweden) or p17 (4C9, ARP342 obtained from R.B. Ferns and R.S. Tedder, Middlesex Hospital Medical School, London, UK) obtained through the National Institute for Biological Standards and Control Centralised Facility for AIDS Reagents (South Mimms, Hertfordshire, UK), followed a rabbit anti-mouse bridging antibody (DakoCytomation, Ely, UK), and 5 nm PAG. Alternatively, sections were stained with mouse polyclonal antibodies against BST2 or double stained with rabbit anti-BST2 and mouse anti-CD63 (antibody 1B5, see [81]). Sections were embedded in uranyl acetate in methylcellulose, as described [82], and examined with a Technai G2 Spirit transmission electron microscope (FEI Company UK. Ltd., Cambridge, UK). Digital images were recorded with a Morada 11 MegaPixel TEM camera (Olympus Soft Imaging Solutions) and the AnalySIS software package. Images were adjusted for brightness and contrast, and figures were assembled with Photoshop CS.

### Flow cytometry analysis

Forty-eight hours after siRNA transfection, non-infected cells were harvested by scraping, washed twice in cold PBS/1% (w/v) BSA and stained for 1 h at 4°C with mouse monoclonal anti-BST2 antibody (Abcam) or control mouse IgG1 (BD Biosciences) in PBS/1% BSA. The cells were then washed three times in PBS/1% BSA, and stained for 1 h at 4°C with an Alexa 647-conjugated donkey anti-mouse antibody in PBS/1% BSA. Cells were washed and fixed in 4% PFA before analysis using the Cytomics FC500 Flow Cytometer (Beckman Coulter).

Alternatively, cells were transfected with siRNAs and, after 48 h, infected with NL4-3 (WT or Udel) HIV-1 pseudotyped with VSV-G at a MOI of 0.5. Twenty-four hours after infection, cells were harvested by scraping, washed twice in cold PBS/1% BSA and stained for 1 h at 4°C with mouse monoclonal anti-BST2 antibody (Abcam) or control mouse IgG1 (BD Biosciences), together with the human anti-Env antibody (2G12) in PBS/1% BSA. The cells were washed three times in PBS/1% BSA, and stained for 1 h at 4°C with Alexa 647-conjugated donkey anti-mouse and PE-conjugated donkey anti-human antibodies in PBS/1% BSA. Cells were washed and fixed in 4% PFA before analysis using the Cytomics FC500 Flow Cytometer. Data acquisition was performed at the Cochin Immunobiology Facility. Gates for PE were set using non-infected cells.

### Analysis of BST2 turnover

Forty-eight hours after siRNA transfection, cells were pre-incubated for 45 min with DMEM with 10 mM Hepes, 1 mg/ml BSA (Calbiochem, VWR, France) and 10 µg/ml cycloheximide (Calbiochem). The cells were then washed and incubated for the indicated times in regular growth medium supplemented with 10 mM Hepes, 10 µg/ml cycloheximide, and 150 ng/ml Epidermal Growth Factor (EGF) (Invitrogen, France), where indicated. The cells were harvested, washed in PBS, lysed and analyzed by western-blotting as described before.

## Supporting Information

Figure S1 **Analysis of Rab proteins depletion.** **(A)** Schematic representation of the Rab sequences targeted by commercial pool siRNAs and designed siRNAs (Rab7A, Rab7A-2 and Rab9A). Black bars correspond to the Rab ORF, and blue, green, red and yellow bars correspond to the positions of the siRNA-targeted sequences. Rab1A siRNA pool from Dharmacon (5’CAGCAUGAAUCCCGAAUAU; 5’GUAGAACAGUCUUUCAUGA; 5’GGAAACCAGUGCUAAGAAU; 5’UGAGAAGUCCAAUGUUAAA) targeted Rab1A mRNA, respectively at positions 392–410, 867–885, 839–857, 941–959. Rab4A siRNA pool from Dharmacon (5’GAAAGAAUGGGCUCAGGUA**;** 5’GUUAACAGAUGCCCGAAUG**;** 5’UUAGAAGCCUCCAGAUUUG**;** 5’UACAAUGCGCUUACUAAUU) targeted Rab4A mRNA respectively at positions 764–782, 529–547, 623–641 and 509–527. Rab5A siRNA pool from Dharmacon (5’GCAAGCAAGUCCUAACAUU**;** 5’GGAAGAGGAGUAGACCUUA**;** 5’AGGAAUCAGUGUUGUAGUA**;** 5’GAAGAGGAGUAGACCUUAC) targeted Rab5A mRNA respectively at positions 736–754, 962–979, 999–1019 and 963–980. Rab6A siRNA pool from Dharmacon (5’GAGAAGAUAUGAUUGACAU**;** 5’GAGCAACCAGUCAGUGAAG**;** 5’AAGCAGAGAAGAUAUGAUU**;** 5’CCAAAGAGCUGAAUGUUAU) targeted Rab6A mRNA respectively at positions 1075–1093, 1113–1131, 1070–1088 and 955–973. Rab7A siRNA (5’GGGAGUUCUGGAGUCGGGAA) and Rab7A-2 siRNA (5’CCACAAUAGGAGCUGACUU3’) targeted Rab7A mRNA, respectively, at positions 274–294 and 348–366. Rab8A siRNA pool from Dharmacon (5’GAAUUAAACUGCAGAUAUG**;** 5’GAACAAGUGUGAUGUGAAU**;** 5’GAACUGGAUUCGCAACAUU**;** 5’GAAGACCUGUGUCCUGUUC) targeted Rab8A mRNA respectively at positions 389–407, 582–600, 522–540 and 282–300. Rab9A siRNA (5’CGGCAGGTGTCTACAGAAG) targeted Rab9A mRNA at positions 573–591. Rab11A siRNA pool from Dharmacon (5’GGAGUAGAGUUUGCAACAA**;** 5’GUAGGUGCCUUAUUGGUUU**;** 5’GCAACAAUGUGGUUCCUAU**;** 5’CAAGAGCGAUAUCGAGCUA) targeted Rab11A mRNA respectively at positions 261–279, 381–399, 703–721 and 336–354. **(B)** HeLa P4-R5 MAGI cells were transfected with siRNAs against luciferase (siLuc) or the different Rab proteins (siRab). Cells lysates were separated by SDS/PAGE and subjected to western blotting with rabbit anti-Rab1A, goat anti-Rab4A, rabbit anti-Rab5A, rabbit anti-Rab6A, mouse anti-Rab7A, goat anti-Rab8A, mouse anti-Rab9A, rabbit anti-Rab11A, or mouse anti-tubulin (as a loading control) antibodies.
(TIF)

Figure S2 **Impact of Rab7A depletion on HIV-1 entry.** HeLa P4-R5 MAGI cells transfected with either control siRNA Luciferase (siLuc) or siRNAs targeting Rab7A protein (siRab7A) were infected with NL4-3 HIV-1 (HIV-1) **(A)** or with NL4-3 HIV-1 pseudotyped with VSV-G (VSV-G HIV-1) **(B)** at a low MOI = 0.005. HIV-1 entry corresponds to the number of infected blue cells counted in siRNA-treated HeLa P4-R5 MAGI cells after one round of infection. This experiment is representative of 6 experiments performed in duplicate. Bars represent the mean of the number of infected cells ± SD from duplicates in one experiment.
(TIF)

Figure S3 **Impact of Rab7A depletion on virus release and genomic RNA encapsidation into particles.** **(A-C)** HeLa cells transfected with either siRNA control Luciferase (siLuc) or siRNA targeting Rab7A proteins (siRab7A and siRab7A-2) were infected with the VSV-G-pseudotyped HIV-1. Viruses and producer cells were collected. This experiment is representative of 3 independent experiments done in duplicate. HIV-1 CAp24 released into the supernatant of the infected cells (Released CAp24, grey graphs bars) **(A)** and present within the cells (Cell-associated CAp24, black graph bars) **(B)** were measured by ELISA quantification. **(C)** HIV-1 Infectivity corresponds to the ratio of the titre of produced virus to the quantity of released CAp24 (Infectious units/µg CAp24). HIV-1 infectivity values obtained for Rab-depleted cells were normalized to those obtained for the control cells, set as 100%. Bars represent the mean ± SD from duplicates of one experiment. RNA was extracted from the cell **(E)** or virus samples **(D)** and subjected to reverse transcription followed by real-time PCR. Unspliced viral genomic mRNAs (gRNA) were selectively amplified using La8.1 and La9 primers. Values obtained for viral genomic RNA were determined relative to those obtained for GAPDH amplification and then normalized to those obtained for the control cells, set as 100%. As a negative control, an equivalent amount of RNA was used to perform the identical reaction in the absence of M-MLV RT (RT control). **(F)** Viral RNA extracted from each viral sample was normalized to the amount of CAp24 released and is shown relative to the RNA from the control cells, set as 100%. Bars represent the means of the percentages ± SD from duplicates of one experiment.
(TIF)

Figure S4 **Most of the HIV particles at the surface of Rab7A knockdown cells have a mature morphology.** HeLa cells transfected with siRNA targeting Rab7A (siRab7A) were infected with VSV-G-pseudotyped HIV-1 for 24 h and then fixed and processed for EM immunolabelling. **(A)** Ultrathin cryosections were double labelled for Env with 10 nm protein A-gold particles and CAp24 and 5 nm protein A-gold. The immunogold labelling allows identification of virus particles and distinguishes viruses from microvilli (MV) that have a similar diameter. CAp24 labelling is also seen on particles with immature morphology (arrows). These particles have a slightly larger diameter and smooth circular outline, with a thick layer of oligomerized Gag underneath the viral membrane. **(B, C, D)** Ultrathin cryosections were double labelled for Env with 10 nm protein A-gold particles and MAp17 and 5 nm protein A-gold. **(B)** Viruses budding from the surface of a cell. As the antibody 4C9 only binds the cleaved mature MAp17, immature virus particles and buds (arrows) remain generally unlabelled. By contrast, mature HIV particles are strongly stained for MAp17 (marked V). **(C, D)** Most of the clustered virus particles at the cell surface are strongly labelled for MAp17, indicating that they are mature, tethered virions. Scale bars = 200 nm.
(TIF)

Figure S5 **Effect of over-expression of the inactive form of Rab7A, T22N, on BST2 turnover and distribution.** HeLa cells were transfected with either wild type Rab7A (GFP-Rab7A WT) or the inactive form of Rab7A (GFP-Rab7A T22N). **(A)** Forty-eight hours after transfection, cells were incubated with cycloheximide (10 µg/ml) for 0 or 4 hours. Cells were lysed and equivalent amounts of each sample (50 µg of protein) were analyzed using western blotting with antibodies against BST2, GFP and tubulin as a loading control. This experiment is representative of 3 independent experiments. **(B)** Forty-eight hours after transfection, cells were permeabilized before fixation and stained with mouse polyclonal anti-BST2 and Alexa 594-conjugated donkey anti-mouse antibodies. Scale bars  = 20 µm.
(TIF)

Figure S6 **Effect of Rab7A silencing on MAp17 and BST2 co-distribution.** HeLa cells transfected with either siRNA control Luciferase (siLuc) or siRNA targeting Rab7A (siRab7A) were infected with VSV-G pseudotyped wt NL4-3 HIV-1 (HIV-1 WT) viruses. **(A)** Twenty-four hours after infection, cells were stained at 4°C with rabbit polyclonal BST2 antibodies and appropriate fluorophore-conjugated secondary antibodies. Cells were then fixed with PFA, permeabilized, labelled with mouse monoclonal MAp17 antibodies and DAPI. Infected cells are indicated with white stars. Scale bars  = 20 µm. **(B)** MA-BST2 colocalization was assessed by calculating the Pearson correlation coefficient on twenty images per condition using the JACoP plugin on ImageJ.
(TIF)
