# Supplementary figures and images for: Rab7A Is Required for Efficient Production of Infectious HIV-1
Source: PLoS Pathog. 2011 Nov 3;7(11):e1002347. doi: 10.1371/journal.ppat.1002347 (PMC3207927; doi:10.1371/journal.ppat.1002347)

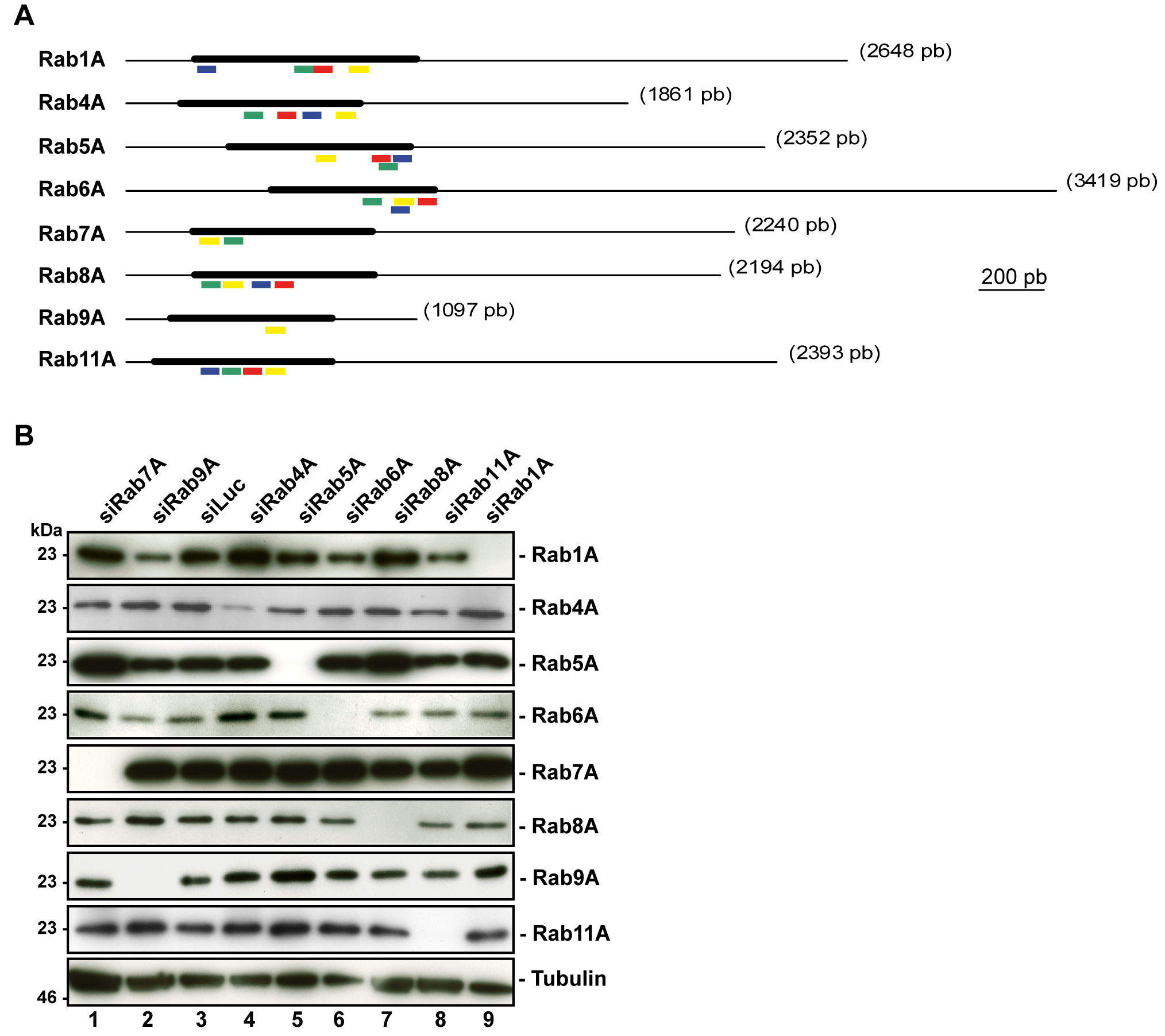

Supplement: Figure S1 — Analysis of Rab proteins depletion. (A) Schematic representation of the Rab sequences targeted by commercial pool siRNAs and designed siRNAs (Rab7A, Rab7A-2 and Rab9A). Black bars correspond to the Rab ORF, and blue, green, red and yellow bars correspond to the positions of the siRNA-targeted sequences. Rab1A siRNA pool from Dharmacon (5’CAGCAUGAAUCCCGAAUAU; 5’GUAGAACAGUCUUUCAUGA; 5’GGAAACCAGUGCUAAGAAU; 5’UGAGAAGUCCAAUGUUAAA) targeted Rab1A mRNA, respectively at positions 392–410, 867–885, 839–857, 941–959. Rab4A siRNA pool from Dharmacon (5’GAAAGAAUGGGCUCAGGUA; 5’GUUAACAGAUGCCCGAAUG; 5’UUAGAAGCCUCCAGAUUUG; 5’UACAAUGCGCUUACUAAUU) targeted Rab4A mRNA respectively at positions 764–782, 529–547, 623–641 and 509–527. Rab5A siRNA pool from Dharmacon (5’GCAAGCAAGUCCUAACAUU; 5’GGAAGAGGAGUAGACCUUA; 5’AGGAAUCAGUGUUGUAGUA; 5’GAAGAGGAGUAGACCUUAC) targeted Rab5A mRNA respectively at positions 736–754, 962–979, 999–1019 and 963–980. Rab6A siRNA pool from Dharmacon (5’GAGAAGAUAUGAUUGACAU; 5’GAGCAACCAGUCAGUGAAG; 5’AAGCAGAGAAGAUAUGAUU; 5’CCAAAGAGCUGAAUGUUAU) targeted Rab6A mRNA respectively at positions 1075–1093, 1113–1131, 1070–1088 and 955–973. Rab7A siRNA (5’GGGAGUUCUGGAGUCGGGAA) and Rab7A-2 siRNA (5’CCACAAUAGGAGCUGACUU3’) targeted Rab7A mRNA, respectively, at positions 274–294 and 348–366. Rab8A siRNA pool from Dharmacon (5’GAAUUAAACUGCAGAUAUG; 5’GAACAAGUGUGAUGUGAAU; 5’GAACUGGAUUCGCAACAUU; 5’GAAGACCUGUGUCCUGUUC) targeted Rab8A mRNA respectively at positions 389–407, 582–600, 522–540 and 282–300. Rab9A siRNA (5’CGGCAGGTGTCTACAGAAG) targeted Rab9A mRNA at positions 573–591. Rab11A siRNA pool from Dharmacon (5’GGAGUAGAGUUUGCAACAA; 5’GUAGGUGCCUUAUUGGUUU; 5’GCAACAAUGUGGUUCCUAU; 5’CAAGAGCGAUAUCGAGCUA) targeted Rab11A mRNA respectively at positions 261–279, 381–399, 703–721 and 336–354. (B) HeLa P4-R5 MAGI cells were transfected with siRNAs against luciferase (siLuc) or the different Rab proteins (siRab). Cells lysates were separated by SDS/PAGE and subjected to western blotting with [file ppat.1002347.s001.tif]

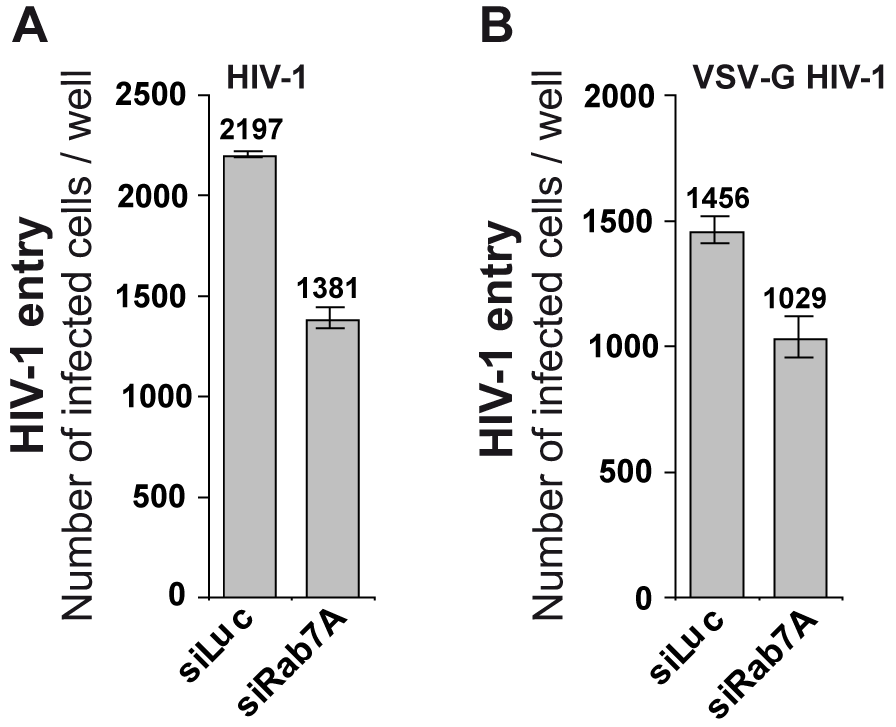

Supplement: Figure S2 — Impact of Rab7A depletion on HIV-1 entry. HeLa P4-R5 MAGI cells transfected with either control siRNA Luciferase (siLuc) or siRNAs targeting Rab7A protein (siRab7A) were infected with NL4-3 HIV-1 (HIV-1) (A) or with NL4-3 HIV-1 pseudotyped with VSV-G (VSV-G HIV-1) (B) at a low MOI = 0.005. HIV-1 entry corresponds to the number of infected blue cells counted in siRNA-treated HeLa P4-R5 MAGI cells after one round of infection. This experiment is representative of 6 experiments performed in duplicate. Bars represent the mean of the number of infected cells ± SD from duplicates in one experiment. (TIF) [file ppat.1002347.s002.tif]

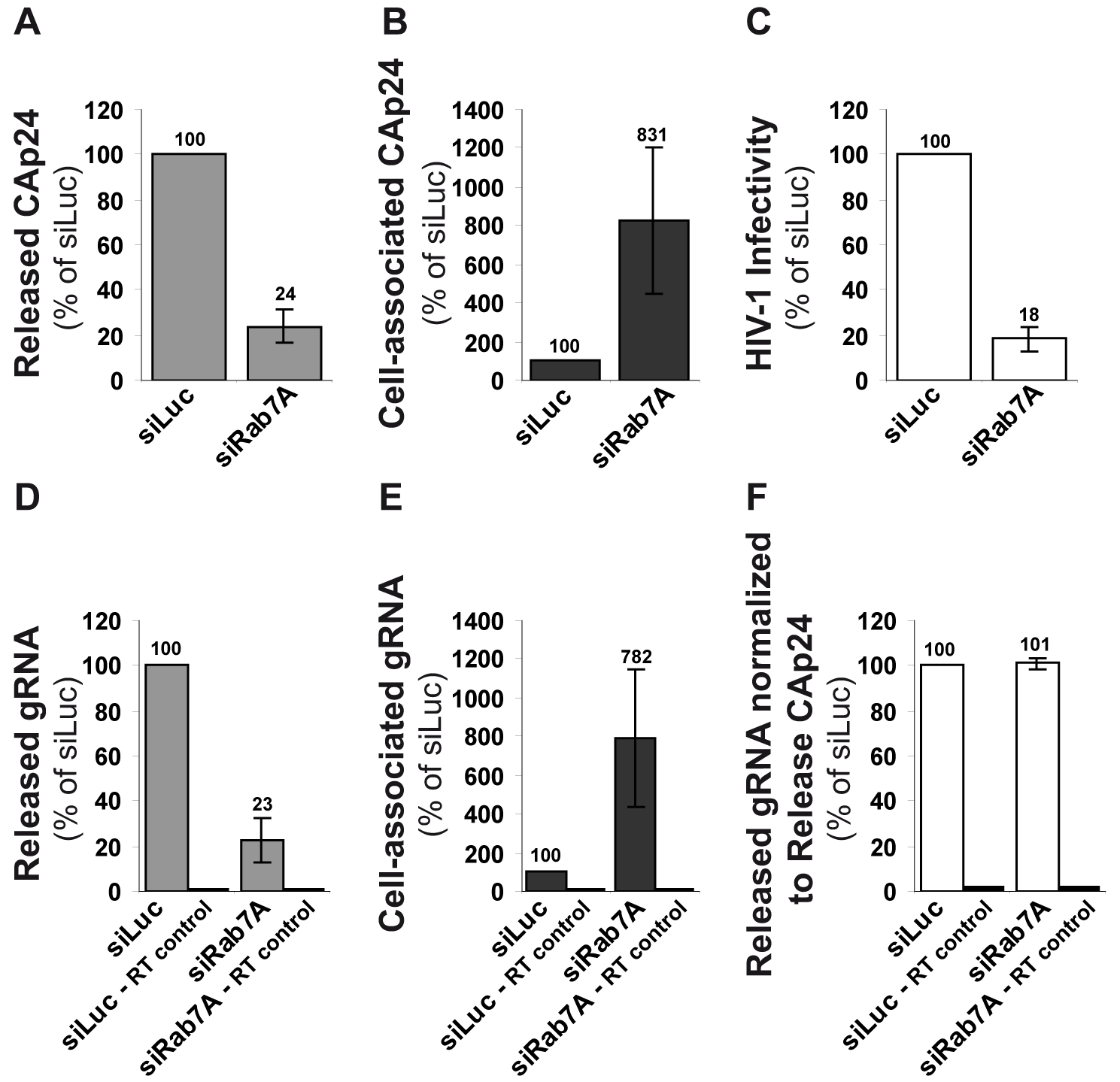

Supplement: Figure S3 — Impact of Rab7A depletion on virus release and genomic RNA encapsidation into particles. (A-C) HeLa cells transfected with either siRNA control Luciferase (siLuc) or siRNA targeting Rab7A proteins (siRab7A and siRab7A-2) were infected with the VSV-G-pseudotyped HIV-1. Viruses and producer cells were collected. This experiment is representative of 3 independent experiments done in duplicate. HIV-1 CAp24 released into the supernatant of the infected cells (Released CAp24, grey graphs bars) (A) and present within the cells (Cell-associated CAp24, black graph bars) (B) were measured by ELISA quantification. (C) HIV-1 Infectivity corresponds to the ratio of the titre of produced virus to the quantity of released CAp24 (Infectious units/µg CAp24). HIV-1 infectivity values obtained for Rab-depleted cells were normalized to those obtained for the control cells, set as 100%. Bars represent the mean ± SD from duplicates of one experiment. RNA was extracted from the cell (E) or virus samples (D) and subjected to reverse transcription followed by real-time PCR. Unspliced viral genomic mRNAs (gRNA) were selectively amplified using La8.1 and La9 primers. Values obtained for viral genomic RNA were determined relative to those obtained for GAPDH amplification and then normalized to those obtained for the control cells, set as 100%. As a negative control, an equivalent amount of RNA was used to perform the identical reaction in the absence of M-MLV RT (RT control). (F) Viral RNA extracted from each viral sample was normalized to the amount of CAp24 released and is shown relative to the RNA from the control cells, set as 100%. Bars represent the means of the percentages ± SD from duplicates of one experiment. (TIF) [file ppat.1002347.s003.tif]

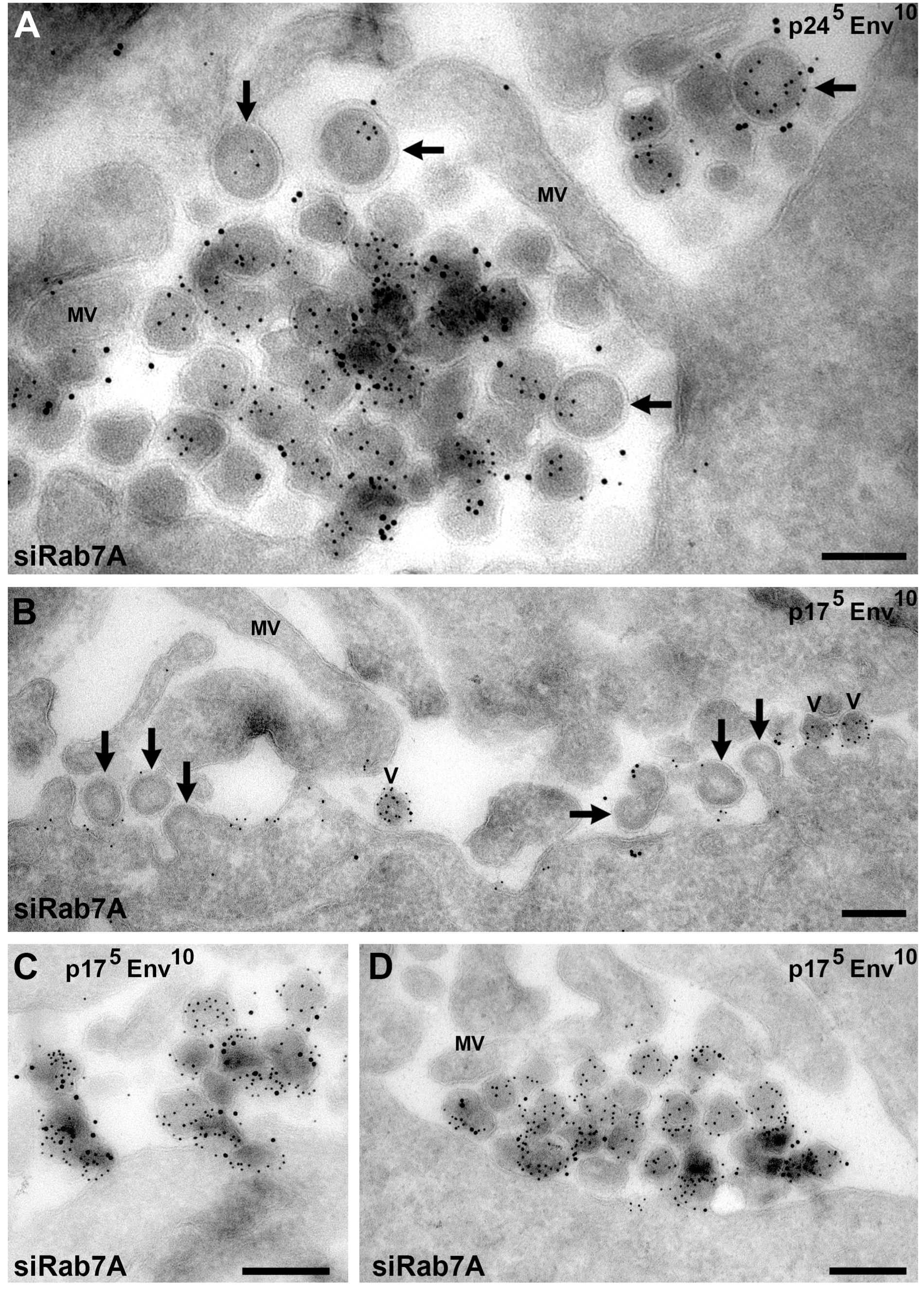

Supplement: Figure S4 — Most of the HIV particles at the surface of Rab7A knockdown cells have a mature morphology. HeLa cells transfected with siRNA targeting Rab7A (siRab7A) were infected with VSV-G-pseudotyped HIV-1 for 24 h and then fixed and processed for EM immunolabelling. (A) Ultrathin cryosections were double labelled for Env with 10 nm protein A-gold particles and CAp24 and 5 nm protein A-gold. The immunogold labelling allows identification of virus particles and distinguishes viruses from microvilli (MV) that have a similar diameter. CAp24 labelling is also seen on particles with immature morphology (arrows). These particles have a slightly larger diameter and smooth circular outline, with a thick layer of oligomerized Gag underneath the viral membrane. (B, C, D) Ultrathin cryosections were double labelled for Env with 10 nm protein A-gold particles and MAp17 and 5 nm protein A-gold. (B) Viruses budding from the surface of a cell. As the antibody 4C9 only binds the cleaved mature MAp17, immature virus particles and buds (arrows) remain generally unlabelled. By contrast, mature HIV particles are strongly stained for MAp17 (marked V). (C, D) Most of the clustered virus particles at the cell surface are strongly labelled for MAp17, indicating that they are mature, tethered virions. Scale bars = 200 nm. (TIF) [file ppat.1002347.s004.tif]

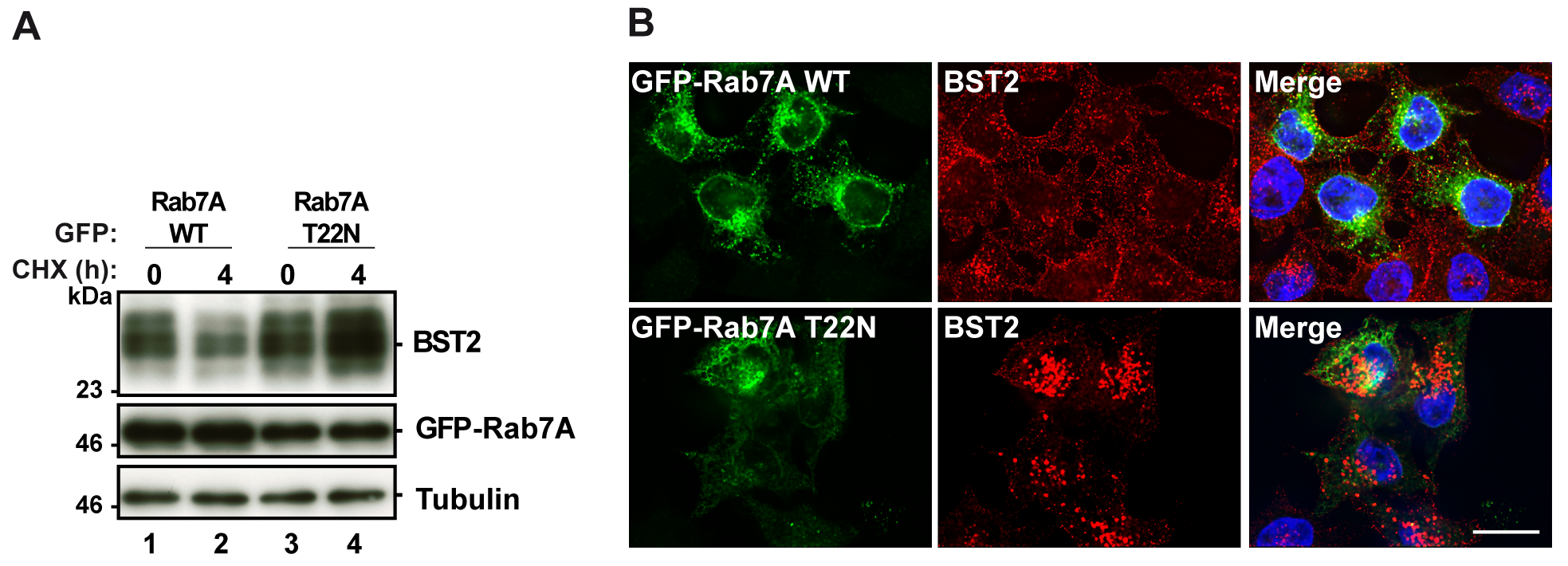

Supplement: Figure S5 — Effect of over-expression of the inactive form of Rab7A, T22N, on BST2 turnover and distribution. HeLa cells were transfected with either wild type Rab7A (GFP-Rab7A WT) or the inactive form of Rab7A (GFP-Rab7A T22N). (A) Forty-eight hours after transfection, cells were incubated with cycloheximide (10 µg/ml) for 0 or 4 hours. Cells were lysed and equivalent amounts of each sample (50 µg of protein) were analyzed using western blotting with antibodies against BST2, GFP and tubulin as a loading control. This experiment is representative of 3 independent experiments. (B) Forty-eight hours after transfection, cells were permeabilized before fixation and stained with mouse polyclonal anti-BST2 and Alexa 594-conjugated donkey anti-mouse antibodies. Scale bars = 20 µm. (TIF) [file ppat.1002347.s005.tif]

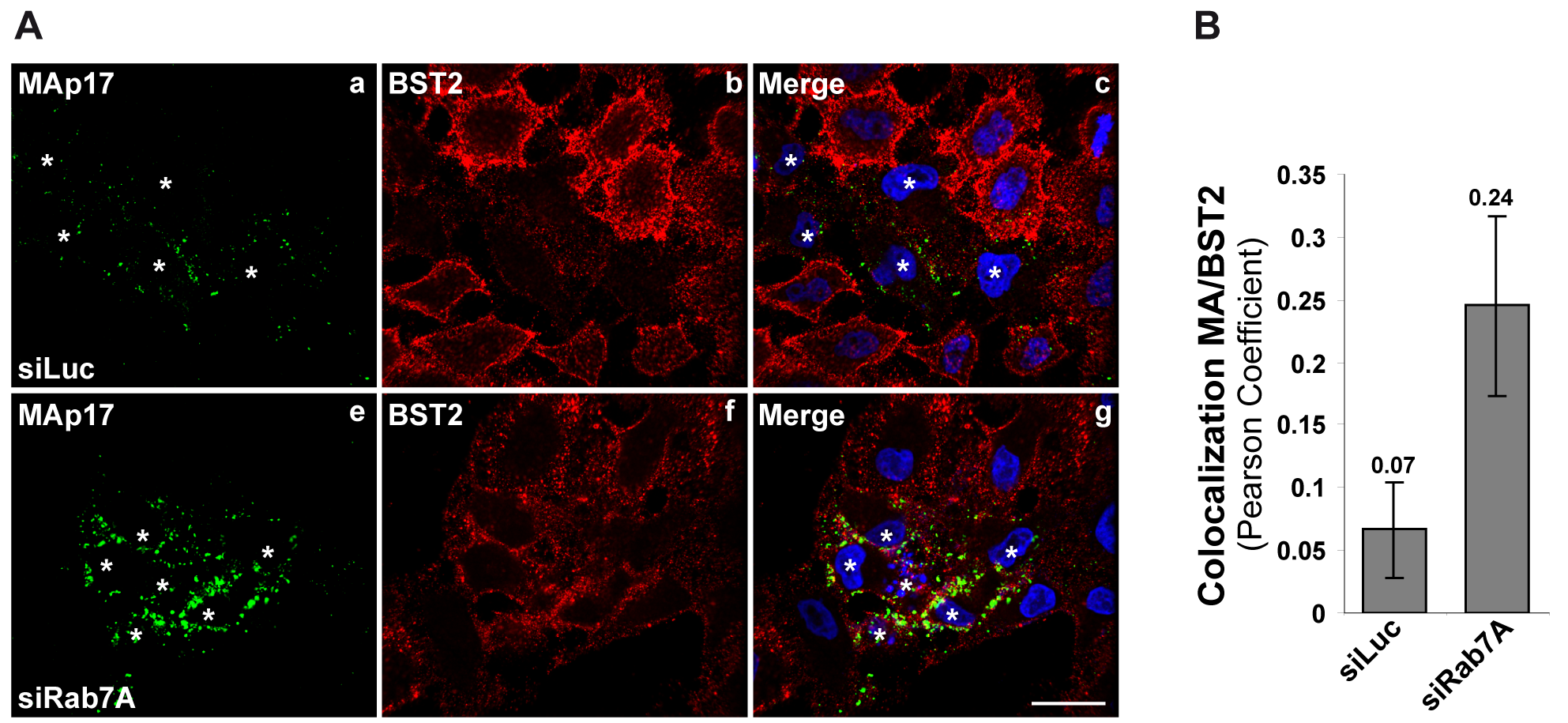

Supplement: Figure S6 — Effect of Rab7A silencing on MAp17 and BST2 co-distribution. HeLa cells transfected with either siRNA control Luciferase (siLuc) or siRNA targeting Rab7A (siRab7A) were infected with VSV-G pseudotyped wt NL4-3 HIV-1 (HIV-1 WT) viruses. (A) Twenty-four hours after infection, cells were stained at 4°C with rabbit polyclonal BST2 antibodies and appropriate fluorophore-conjugated secondary antibodies. Cells were then fixed with PFA, permeabilized, labelled with mouse monoclonal MAp17 antibodies and DAPI. Infected cells are indicated with white stars. Scale bars = 20 µm. (B) MA-BST2 colocalization was assessed by calculating the Pearson correlation coefficient on twenty images per condition using the JACoP plugin on ImageJ. (TIF) [file ppat.1002347.s006.tif]
